# Substantial impact of mobility restrictions on reducing COVID-19 incidence in Italy in 2020

**DOI:** 10.1093/jtm/taac081

**Published:** 2022-07-24

**Authors:** Marco Vinceti, Erica Balboni, Kenneth J Rothman, Sergio Teggi, Stefania Bellino, Patrizio Pezzotti, Fabrizio Ferrari, Nicola Orsini, Tommaso Filippini

**Affiliations:** Environmental, Genetic and Nutritional Epidemiology Research Center (CREAGEN), Section of Public Health, Department of Biomedical, Metabolic and Neural Sciences, University of Modena and Reggio Emilia, 41125 Modena, Italy; Department of Epidemiology, Boston University School of Public Health, Boston, MA 02118, USA; Environmental, Genetic and Nutritional Epidemiology Research Center (CREAGEN), Section of Public Health, Department of Biomedical, Metabolic and Neural Sciences, University of Modena and Reggio Emilia, 41125 Modena, Italy; Department of Epidemiology, Boston University School of Public Health, Boston, MA 02118, USA; RTI Health Solutions, Research Triangle Park, NC 27709, USA; Department of Engineering ‘Enzo Ferrari’, University of Modena and Reggio Emilia, 41125 Modena, Italy; Department of Infectious Diseases, Italian National Institute of Health, 00161 Rome, Italy; Department of Infectious Diseases, Italian National Institute of Health, 00161 Rome, Italy; TerrAria srl, 20125 Milan, Italy; Department of Global Public Health, Karolinska Institute, Stockholm, 11365 Stockholm, Sweden; Environmental, Genetic and Nutritional Epidemiology Research Center (CREAGEN), Section of Public Health, Department of Biomedical, Metabolic and Neural Sciences, University of Modena and Reggio Emilia, 41125 Modena, Italy; School of Public Health, University of California Berkeley, 1995 University Avenue, Berkeley, CA 94704, USA

**Keywords:** COVID-19, SARS-CoV-2, outbreak, epidemiology, cell phone, lockdown, peak, means of transport, mobility

## Abstract

**Background:**

Italy was the first country after China to be severely affected by the COVID-19 pandemic, in early 2020. The country responded swiftly to the outbreak with a nationwide two-step lockdown, the first one light and the second one tight. By analyzing 2020 national mobile phone movements, we assessed how lockdown compliance influenced its efficacy.

**Methods:**

We measured individual mobility during the first epidemic wave with mobile phone movements tracked through carrier networks, and related this mobility to daily new SARS-CoV-2 infections, hospital admissions, intensive care admissions and deaths attributed to COVID-19, taking into account reason for travel (work-related or not) and the means of transport.

**Results:**

The tight lockdown resulted in an 82% reduction in mobility for the entire country and was effective in swiftly curbing the outbreak as indicated by a shorter time-to-peak of all health outcomes, particularly for provinces with the highest mobility reductions and the most intense COVID-19 spread. Reduction of work-related mobility was accompanied by a nearly linear benefit in outbreak containment; work-unrelated movements had a similar effect only for restrictions exceeding 50%. Reduction in mobility by car and by airplane was nearly linearly associated with a decrease in most COVID-19 health outcomes, while for train travel reductions exceeding 55% had no additional beneficial effects. The absence of viral variants and vaccine availability during the study period eliminated confounding from these two sources.

**Conclusions:**

Adherence to the COVID-19 tight lockdown during the first wave in Italy was high and effective in curtailing the outbreak. Any work-related mobility reduction was effective, but only high reductions in work-unrelated mobility restrictions were effective. For train travel, there was a threshold above which no further benefit occurred. These findings could be particular to the spread of SARS-CoV-2, but might also apply to other communicable infections with comparable transmission dynamics.

## Introduction

To counteract the spread of an airborne disease such as COVID-19 in the absence of effective therapies and a vaccine, public authorities rely on non-pharmacological control measures. The most widely used, though controversial, measure has been the adoption of legal means to decrease social interaction through advised or mandatory mobility reductions. These measures were described as ‘lockdowns’ when they were stringent enough to curtail the daily activity of most individuals.^1–3^ Imposition of lockdowns has dramatic psychological effects as well as adverse economic effects. Furthermore, considerable debate has swirled about its efficacy relative to alternative means to counteract the spread of COVID-19.^3–6^

Italy was the first country after China to experience a severe COVID-19 outbreak, beginning 20 February 2020, the date of the first diagnosed case. Soon after, the outbreak swept throughout the country, especially Northern Italy. National authorities imposed two lockdowns, a limited one on February 23 (hereafter named ‘light lockdown’) and a tight one on March 8 (‘tight lockdown’).^3^^,^^7^ The latter measure imposed rigid legal constraints on mobility to decrease social interactions. Following this unprecedented measure, by April of 2020 the surge in COVID-19 cases, hospital admissions and deaths were curbed and then reversed. Lockdown was terminated on 4 May 2020, with the end of mobility restrictions and the progressive reopening of all activities and movements.

Italy has a tradition of intense social interactions. It was ranked second in the world in a recent review and meta-analysis of social contact patterns and related implications for communicable diseases.^8^ For this reason, the feasibility of a tight lockdown was questioned. Furthermore, its efficacy was considered dubious by some scientists and public health authorities.^9–11^ We aimed to investigate these issues and extend the findings of a previous study, by using all the 2020 data of an extremely accurate indicator of individual mobility, mobile phone tracking.^3^^,^^12^^,^^13^ Italy showed in 2020 a high smartphone penetration,^14^ making mobile phone tracking data a reliable indicator of mobility. The first COVID-19 wave presented a research opportunity because there were fewer factors that might influence the outcome, such as region-specific non-pharmacological interventions that were adopted since November 2020, spread of viral variants identified only since September 2020,^15^ availability of vaccine (which became available in late December 2020), and possible occurrence of immunity from prior waves of the outbreak.^16^ To assess lockdown compliance and efficacy, we used mobile phone tracking to trace mobility patterns^3^^,^^12^^,^^13^^,^^17^ and analyzed the effect of restricting specific types of mobility, according to their underlying reasons and the means of transport.

## Methods

### Study period and data

We used health data and cell phone mobility data, by extending a previous analysis geographically (from three regions to all of Italy) and over time (to the entire COVID-19 first wave).^3^ We also added three COVID-19 endpoints and details about reason and means of travel. The time period we studied corresponded to the first COVID-19 wave in Italy, during the first half of 2020.^3^^,^^18^ Soon after the first case of COVID-19 in Italy was identified on 20 February 2020, the Government issued a nationwide light lockdown, on February 23. The February 23 decree issuing the light lockdown gave public and health authorities the legal wherewithal to decrease mobility of people and goods throughout Italy. This decree allowed for closing down private activities as well as public, including schools, in any area that was even minimally affected by the pandemic. It also included locking down the residents of a few highly affected municipalities in the Lombardy and Veneto regions (the so-called ‘red zones’ of Codogno, Vò Euganeo, etc.). However, all Northern Italy regions, regardless of the pandemic reach, agreed simultaneously to apply some of these restrictions to decrease social interactions. These steps included closure of schools and universities, reduction of public activities, gatherings in churches, museums, and leisure areas, and reductions in use of public transportation. Strict control on incoming persons into the country from countries at high risk, and isolation of infected individuals and close contacts were also decreed and implemented. The subsequent lockdown was the tight one and was imposed on March 8 in most of the Veneto, Emilia-Romagna and Lombardy regions, and on March 9 in all remaining Italian regions. This tight lockdown immediately forced all Italian residents to avoid any movements outside their residence, apart from exceptional occupational need or an emergency, all of which required formal documentation. The mobility restrictions allowed only one family member to go to a grocery store, mandated to be the closest shop to the individual’s residence. Subjects infected by SARS-CoV-2 or having been in contact with infected persons were banned from exiting their residence. Individuals not complying with these restrictions were subject to criminal liability. On March 22, the mobility restrictions of the tight lockdown were further strengthened by forbidding the few individuals temporarily staying away from their own municipality to return home, while the ban on any non-essential commercial activity become almost complete. The tight lockdown was terminated on May 4 by ending most mobility restrictions, and a subsequent government decree issued on May 16 soon led to the end of the remaining large-scale mobility restrictions, to the re-opening of almost all activities, and to relaxing requirements for social distancing.

We obtained validated data on health endpoints from the COVID-19 surveillance system at the National Institute of Health,^19^ including validated daily province-specific new cases of SARS-CoV-2 infections and COVID-19 hospital admissions, intensive care unit (ICU) admissions and deaths, overall and by sex and 10-year age group. We used the total daily number of new events occurring in each geographical unit investigated (province, region and country) as the outcome of interest for the present study.

To assess individual mobility, we used data on the 2020 movements of mobile phones in Italy, purchased from ‘Teralytics’ (Zurich, Switzerland) whose mobile phone tracking data have been already used to model COVID-19 transmission in Germany^2^ and the USA.^20^ Mobile phone movements were estimated from mobile signals collected in cell sites, by using the real movements of the mobile phones of the largest company operating in the Italy (WIND-TRE®, 27 million cell phones, nearly 30% of all users in Italy) and by estimating the movements from the remaining mobile phone companies operating in the country, in order to get a picture of the entirety of Italian cell phone traffic. Each phone was identified through the subscriber identification module (SIM), after excluding SIM cards belonging to automatic devices such as alarms (‘machine-to-machine’). The entire national cell phone traffic during the period considered was modelled based on a machine learning algorithm named ‘Matrix’, that took into account cell phone tracking on a geographical and temporal basis and population density at small area levels.^21^ A movement was assumed when the mobile phone switched from one tower to another one (generally in a 500–3000 m range depending on the degree of urbanization), and a ‘dwell time’ within the same base station of at least 30 minutes could be identified both before and after the trip. The anonymized final dataset consisted of a daily trip counter of movements from one cell of departure to another cell of arrival.

Mobile phone tracking allowed us to identify the purpose of travel and the means of transport (road, train or plane) associated with each movement of the smartphone. The classification by trip purpose was based on the second most visited place during the month. Means of transport of each trip were identified based on analysis of the rapidity of the movements of cell phones, the location of motorways, train stations and airports, and the systematic behaviour of sets of cell phones. For instance, a bundle of mobile phones being switched off close to an airport location and being collectively reactivated close to another airport within a short period of time was interpreted as indicating air travel. Available reasons for travelling were categorized as going to work and the return trip (work-related movements), or work-unrelated movements. Movements shorter than 30 km could not be assigned to a specific means of transport by the random-forest algorithm, and were therefore labelled as ‘unclassified’. Movements between different provinces were assigned to the province of departure.

We also retrieved data from several other sources: (i) provincial data about population structure (including overall resident population, single-family homes and elderly index) from the National Institute of Statistics,^18^ (ii) the population-weighted average of ground-level temperature, humidity and ultraviolet radiation from the European Space Agency Copernicus program^22^ and (iii) particulate matter with diameter < 10 μm (PM_10_) from the ENSAMBLE model of the CAMS European air quality forecasts.^23^

### Data analysis

We modelled the province- and region-specific time trends of daily mobility patterns from time-series of mobile phone movements, using a Newey-West linear regression model with heteroskedastic and autocorrelated error structure up to 7 days.^24^ In modelling the mean number of movements as a function of calendar time in days, we used a mix of piecewise constant (degree-0) and linear (degree-1) splines to allow shift in both level and trend at the two major intervention dates (February 23 and March 8), at March 22 when the lockdown was further tightened, and at May 4, when mobility measures started to be eased.

Using the same methodology, we also modelled the time trends in COVID-19 outcomes based on the daily number of new events from each province and region. To do that, we used calendar time in days with restricted cubic splines using knots at 5th, 27.5th, 50th, 72.5th and 95th percentile, to derive the day of peak occurrence. For each of the four COVID-19 outcomes, we defined as ‘time-to-peak’ the number of days elapsed since the start of the tight lockdown, March 8, to the day with the maximum level of the curve. In the second stage, we examined the relation between the proportional reduction in daily movements after the tight lockdown compared with the period before any restrictions were imposed, i.e. on Monday 10 February 2020. The association between proportional reduction in daily movements on March 9 in relation to median time-to-peak, adjusting for population, number of single-family homes, old age index, temperature, humidity, PM_10_ concentration and ultraviolet radiation as potential confounders,^25–30^ was modelled using a restricted cubic spline regression model with knots at the 10th, 50th and 90th percentile. Besides the analysis on the overall dataset, we considered also a subset of urbanized provinces that had at least 50 deaths for COVID-19 for 100 000 residents in the first wave, and a subset of the health outcomes, excluding people aged 70 or above. The first choice was intended to exclude provinces with few SARS-CoV-2 infections and COVID-19 events, where factors other than large scale mobility, such as clusters in nursing homes, hospitals or small social groups, could have dominated the health outcomes. Subgroup analysis with exclusion of people aged 70 and more was intended to focus on age groups characterized by the highest mobility. All analyses were repeated separately for work and non-work-related movements and for the different means of transport, without and with additional adjustment for the other types of transport.

**Figure 1:**
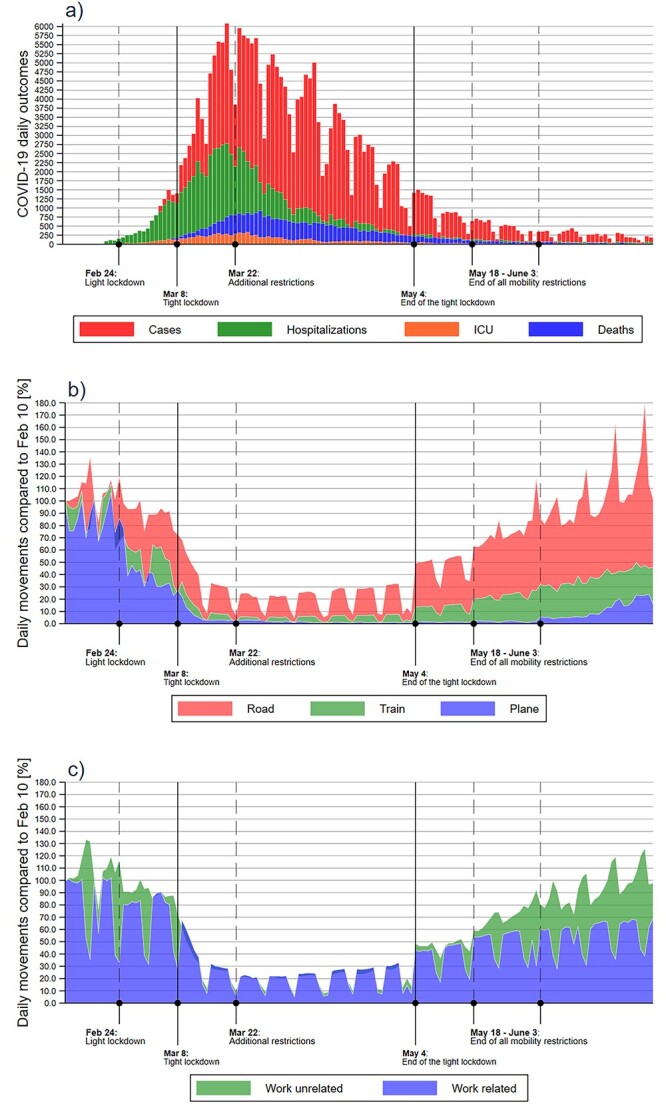
Lockdown timeline and daily number of COVID-19 endpoints (a) and of people movements according to means (b) and reasons (c) of transport in Italy during February–June 2020.

## Results

Figure 1 shows the overall daily number of endpoints of interest (new SARS-CoV-2 infections, COVID-19 related hospital and ICU admissions, and COVID-19 deaths) in Italy and of mobile phone movements according to their type. It also shows the timeline of the key legal and public health interventions in the country with reference to the restriction of mobility and other measures to decrease social interaction. The distribution of the events was skewed to the right, with deaths showing a lag time versus hospital and ICU admissions and new infections. We report in Supplementary Table S1 the overall characteristics of the collected and computed data for the analysis, including cumulative number of events on a geographic basis by 30 June 2020, time-to-peak of these endpoints, mobile phone movements and movement reductions at different time points, and population. Supplementary Figure S1 shows the province-specific cumulative incidence of the four study endpoints at March 9, and the reduction in mobile phone movements as compared with February 10, at three time points (all Mondays): February 24, March 9 and March 23. The reduction in individual movements was 55% nationwide immediately after the tight lockdown (March 9) and 82% two weeks later (March 23). It was much smaller (14%) following the first, lighter lockdown (February 24). The reduction was uneven by region and province, and peaked in the region and the provinces where the outbreak hit hardest, such as Lombardy region and within it the provinces of Milan, Bergamo, Brescia, Cremona and Lodi. These areas are also highly industrialized, and characterized by a large number of motorways and main roads, train stations and airports, and therefore by an extremely high population mobility. The time-to-peak of new cases of SARS-CoV-2 infections from the onset of the tight lockdown was 18 days, with similar values for the most swiftly and severely affected regions of Lombardy, Veneto and Emilia-Romagna, while for COVID-19 deaths, hospital admissions and ICU admissions at the country level the figures were 20, 14 and 15 days, respectively.

Supplementary Tables S2-S3 report the same type of data, stratified by reasons for travel (work-related and work-unrelated) and means of transportation (car, airplane, train and other). After the first light lockdown, the reduction in mobile phone movement related to work (20%) was higher than for other reasons (10%), while after the tighter lockdown work-related movement decreased of 49% (progressing to 79%), while work-unrelated movement of 58% (later 84%). Italy experienced a change in road movements of −4 and −52% immediately after the light and the tight lockdowns, reaching a trough of −82% on March 23, while the corresponding figures were − 26, −84 and − 97% for airplane travel, −29, −79 and − 95% for train travel, and − 9, −39 and − 68% for unclassified movements (Supplementary Table S3).

**Figure 2:**
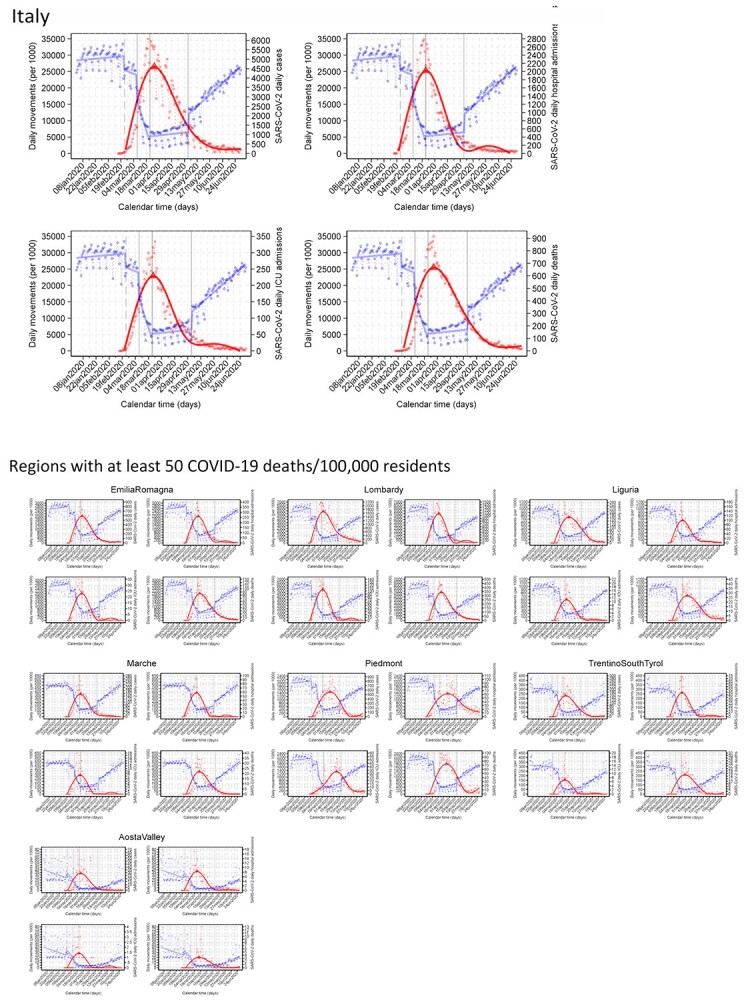
Daily absolute numbers of people movements (blue dots) and COVID-19 events (red dots) in Italy during the first wave and in the seven regions with 50 or more COVID-19 deaths in the first wave of the pandemic. Red line shows the predicted mean number of new COVID-19 cases obtained with restricted cubic splines of calendar days with five knots to identify the maximum predicted value (i.e. day of peak occurrence—red triangle), fitting time-series data using the Newey–West regression model.

In Figure 2, we report cell phone movement patterns during the first wave and the related curves for the different COVID-19 outcomes in Italy and in the seven regions with at least 50 COVID-19 deaths per 100 000 residents (plots for the remaining regions and all provinces available on request). The movements showed a weak decrease after the first lockdown, a more substantial decrease following the second tighter lockdown, and a progressive further decrease over time in the two weeks after the tight lockdown, followed by a cresting and then decline in COVID-19 endpoints. The corresponding plots for the population under 70 years of age showed comparable results, though with considerably fewer events, particularly for COVID-19 deaths.

**Figure 3:**
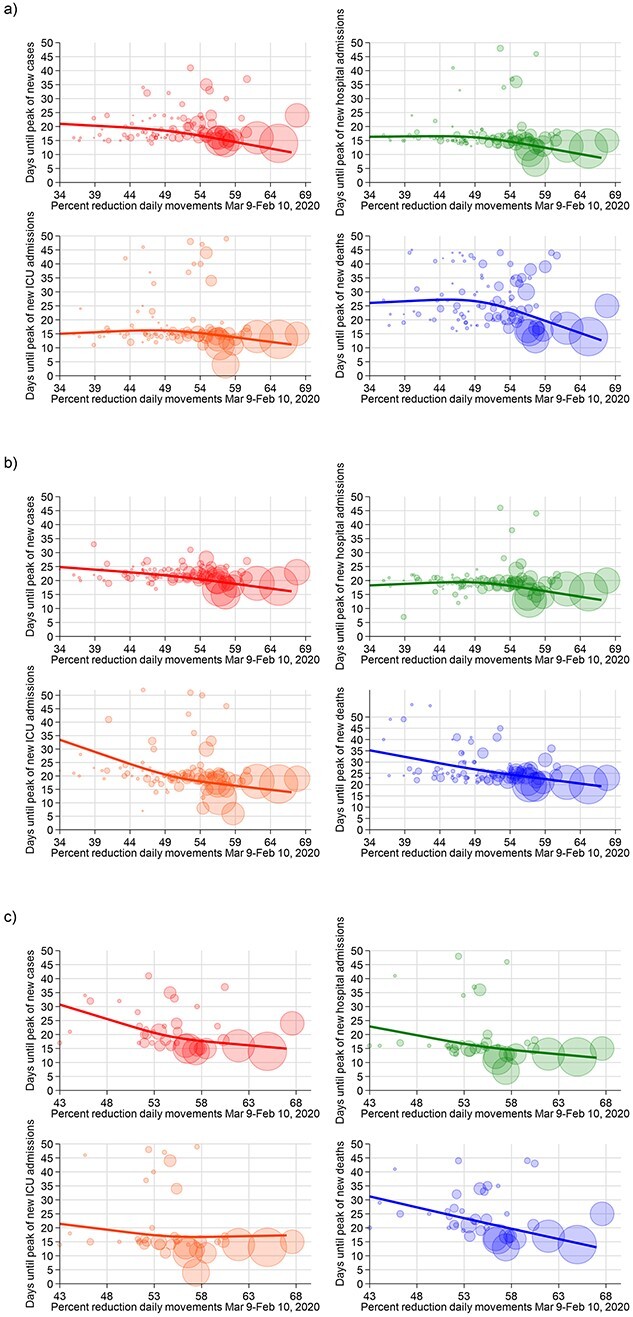
Days until the peak of the COVID-19 endpoints and percent reduction of people movements for provinces in Italy in (a) the entire dataset, (b) for age < 70 and (c) in provinces with cumulative deaths/10^5^ ≥ 50. The solid line shows the results of the spline analysis adjusted for meteorological factors, PM_10_, old age index, population and single-family homes; each bubble, representing a province, has a size proportional to the cumulative number of cases at 8 March 2020.

In regression analyses, strong reductions (≥45%) in daily mobile phone movements in the March 9–February 10 period were almost linearly associated with reduction in time-to-peak of new SARS-CoV-2 infections (Figure 3a). Below this level of reduction in phone movement, the pattern was reversed, although the estimates were based on only a few provinces with the smallest number of cases. The same pattern was seen for ICU admissions, while overall hospital admissions showed such clear reduction only when mobility reduction exceeded 45%. For COVID-19 deaths, we found a U-inverted pattern, since mobility reductions up to 45% showed positive to null associations with time-to-peak, while above this level the sharp decrease in cell phone movements was associated with a much shorter time-to-peak.

When restricting the analysis to the population < 70 years of age, we observed a nearly linear shortening of time-to-peak with decreased mobility across its entire range (Figure 3b), as was also true for provinces with ≥50 deaths/100 000 inhabitants (Figure 3c), and when considering only provinces with ≥200 COVID-19 deaths (Supplementary Figure S2).

We re-run the analysis by removing among the potential confounders either air pollution (PM_10_) or meteorological factors (temperature, humidity and ultraviolet radiation) or both, and we found little if any change in the shape of the regression curves (Supplementary Figure S3).

**Figure 4:**
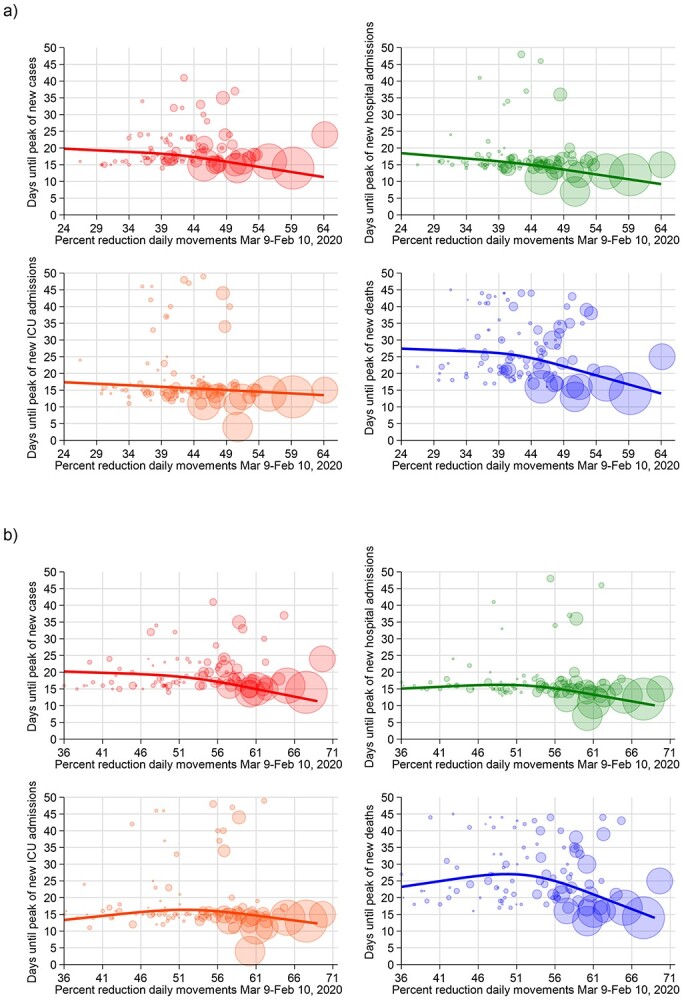
Days until the peak of the COVID-19 endpoints and percent reduction of people movements for provinces in Italy for (a) work-related road movements and (b) work-unrelated movements. The solid line shows the results of the spline analysis adjusted for meteorological factors, PM_10_, old age index, population and single-family homes; each bubble, representing a province, has a size proportional to the cumulative number of cases at 8 March 2020.

We repeated the analyses by stratifying for reasons of travelling and the means of transport. For work-related travel (Figure 4), we found an inverse association between intensity of mobility restrictions and time-to-peak of the COVID-19 endpoints, which was stronger when such restrictions exceeded 40–50%, and almost linear for one of the outcomes, ICU admissions. When instead the analysis focused on mobility restrictions associated with other reasons aside from movement to the workplace, we observed roughly an inverted U-shaped association between reduced mobility and the outcomes, particularly for COVID-19 deaths, with a turning point around the 50% of mobility reductions.

**Figure 5:**
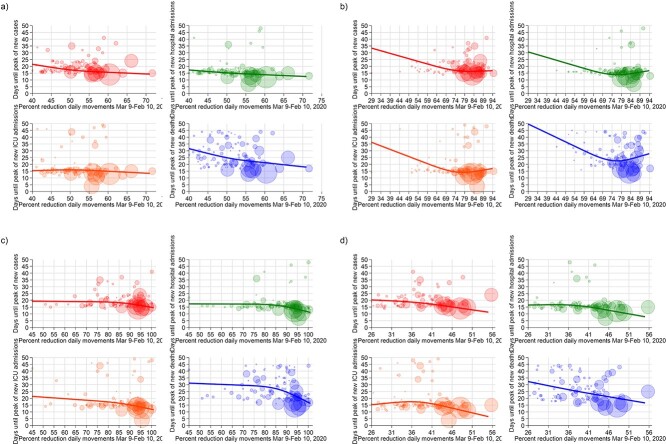
Days until the peak of the COVID-19 endpoints and percent reduction of people movements for provinces in Italy: (a) road movements, (b) train movements, (c) plane movements and (d) unclassified movements (<30 km). The solid line shows the results of the spline analysis adjusted for meteorological factors, PM_10_, old age index, population and single-family homes; each bubble, representing a province, has a size proportional to the cumulative number of cases at 8 March 2020.

With reference to the means of transportation (Figure 5), we found that restricting the mobility to movements by road was associated with a roughly linear decrease in the time-to-peak of the COVID-19 outcomes across the entire range of restrictions, with a steeper decline for deaths. The corresponding patterns for mobility by airplane showed that the highest values of reduction were associated with a substantially stronger decrease in the time-to-peak, especially for the most severe outcomes. In particular, a reduction in mobility by airplane exceeding 85% was accompanied by a drop in COVID-19 deaths. Mobility by train showed a distinctive pattern: the decrease in time-to-peak was almost linear for all outcomes, and steeper for the most severe ones, up to a percentage of reduction of 75%, above which the time-to-peak reduction flattened or even started again to increase. Finally, the reduction in unclassified movements was associated with a shortening in time-to-peak of all outcomes above 40% of reduction apart for COVID-19 deaths, for which the decline occurred at any amount of mobility restriction. By repeating the aforementioned analyses after including all the other types of transport in the regression model to control for possible confounding, results were similar to what was found without adjusting for the other types of transportation (Supplementary Figure S4).

## Discussion

We found that compliance with the tight lockdown in Italy was prompt and diligent, followed by as much as a 90% reduction in mobility in the areas characterized by the highest spread of SARS-CoV-2 infection and COVID-19, and over 80% on a nationwide basis. These figures are higher than those characterizing the behaviour of other European populations under a roughly comparable lockdown scenario (65% for France^31^). Our study showed that a pronounced general limitation of mobility of this Western, highly dynamic and large population, leading to markedly reduced social interactions, was followed by a dramatic decrease in health outcomes that were an objective measurement of the COVID-19 outbreak, i.e. hospital and ICU admissions, and COVID-19 related deaths. An effect was also seen on the count of new SARS-CoV-2 infections, but this outcome, used to assess the effect of public health measures against COVID-19 by many investigators, including ourselves, had substantial limitations. The number of new infections identified depended on the availability of testing, on the policy leading to individual of subgroup testing, and on the organization of the screening and diagnostic programs, with their inherent delays in admitting to testing and releasing the analytical results. On the other hand, these factors do not affect the three most serious COVID-19 outcomes, hospital admissions, ICU admissions and deaths. Considering the incubation period of COVID-19 during its first wave, being around 5–6 days in addition to delays in testing performance and confirmation,^3^ we may assume that the effect of the lockdown was extremely swift, i.e. 10 to 15 days from its implementation, including the effect on the most serious outcomes such as ICU admissions and deaths. This beneficial effect occurred particularly in the areas with the highest reduction in mobility and the most severely hit regions, such as Lombardy region, and provinces such as Bergamo, Cremona, Lodi and Piacenza. A causal connection between mobility reduction and curbing of the outbreak is supported by the absence during the study period of non-pharmacological interventions, effective disease therapy and vaccination. The effect of mobility reduction could be unique to control of SARS-CoV-2, but might also apply to communicable disease from other airborne agents with transmission dynamics comparable with that of SARS-CoV-2. Finally, we note that our study lacked a control community within which no mobility restriction was applied, thus reducing our capacity to characterize the overall effect of lockdown on COVID-19 spread and clinical severity, as compared with the absence of any such public health intervention.

A causal and strong relation between tight lockdown and curbing of COVID-19 is further strengthened by the observation that the findings were substantially unchanged after restricting the analyses to outcomes in people below 70 years of age. This subset of the population has higher mobility and is less susceptible to the most severe outcomes when infected, and was much less affected by specific high-risk environments such as nursing homes and hospital settings. Overall, our findings indicate that a tight lockdown was highly effective in reducing mobility in Italy, and in temporarily curbing the COVID-19 outbreak, thus decreasing the burden of disease until public health measures such as vaccination became available. This assessment appears to be consistent with observations in other Western countries, and contrasts with the smaller effects from mask wearing and less intensive mobility restrictions.^9^^,^^32^^,^^33^

Our analysis also showed that any reduction in work-related mobility had beneficial effects on the COVID-19 outcomes. The pattern for work-unrelated movements was non-linear, indicating that a reduction above 50% was needed to curb the outbreak, at least with reference to the most severe outcomes. For hospital admissions and deaths, no beneficial effect emerged below the 50% approximate threshold, indicating that restrictions up to this amount have little capacity to limit the type of work-unrelated social interactions that lead to disease transmission. The design of our study did not allow us to identify how much of the beneficial effects of restricting work-related movements stemmed from limiting commuting to work, especially through public transportation,^34^ or from avoiding the interactions in the work environment.^35^^,^^36^ Also, the work movement reductions could have been slightly underestimated because of the travel during the working hours (e.g. for bus drivers and couriers) that were not captured as work-related by the classification algorithm.

Contrary to the pattern observed for work-related movements, we found that a high level of restrictions in work-unrelated social interactions must be achieved to curtail outbreak spread and its most serious outcomes, implying that a ‘light’ lockdown, with a mobility reduction <50% outside the work environment, is substantially ineffective to curb the outbreak. Therefore, when facing a severe airborne disease similar to COVID-19 and being forced to use non-pharmacological interventions such as mobility restrictions, the decrease in social interactions for non-professional environments should exceed 50% to achieve satisfactory results and then be increased as much as possible and ‘socially’ sustainable, while any decrease in interactions within work environments is accompanied by public health benefits from limiting spread of the virus.

Concerning the means of transportation, the substantial reduction in road traffic appears to have reduced COVID-19 outcomes, particularly deaths. Benefits were also evident for reduced airplane travel, especially when air traffic was substantially reduced. However, most provinces clustered in the right part of the regression graph (due to the almost entire grounding of commercial aircrafts), making it difficult to measure air travel restrictions. Concerning mobility by train, another key source of concern for transmission of this disease,^37^ our results are of relevance since they indicate that beneficial effects on outbreak spread proportionally occur throughout a broad range of mobility reduction up to a figure of 70%. Above that threshold, however, no further benefit was achieved, thus suggesting that social distancing in the trains at that level of mobility reduction is satisfactory in terms of spread of an airborne disease with the characteristics of COVID-19.^38^^,^^39^ Given that during 2020 some decrease occurred in the overall train traffic in Italy,^40^ around 20% in terms of total person-distance travelled, the approximate reduction in individual train occupation required to achieve the maximal effectiveness in counteracting the spread of COVID-19 can therefore be estimated in the order of 55–60%. Such figure could be considered a public health guideline for safe train occupation (along with face masks) in the presence of communicable disease with the transmissibility pattern characterizing COVID-19.

## Author contributions

M.V., S.B. and T.F. designed the original study, and with K.J.R. and E.B. analyzed and interpreted the data, and drafted the manuscript. N.O. designed the code with T.F. and E.B. E.B. and T.F. carried out data analysis. S.B. and P.P. provided processed health data for analysis, S.T. and F.F. downloaded and processed the environmental data. E.B. and T.F. downloaded and processed cell phone movements and carried out the final data analysis. All authors contributed to, read and approved the final manuscript.

## Supplementary Material

Supplementary_material_JTM_taac081
