# Supplementary material for: Substantial impact of mobility restrictions on reducing COVID-19 incidence in Italy in 2020
Source: J Travel Med. 2022 Jul 24;29(6):taac081. doi: 10.1093/jtm/taac081 (PMC9384467; doi:10.1093/jtm/taac081)
Supplement: Supplementary_material_JTM_taac081 [file supplementary_material_jtm_taac081.docx]

**Supplementary Table 1.** Summary of population size, cumulative number of COVID-19 outcomes by June 30, 2020, time-to-peak of health endpoints, mobile phone movements reductions at different time points, and mobile phone movements on a geographic basis.

| **Province** | **Population** | **Cumulative cases at June 30** | **Cumulative cases at June 30 per 100000 inhabitants** | **Time-to-peak of Sars-Cov-2 infections** | **Cumulative deaths at June 30** | **Cumulative deaths at June 30 per 100000 inhabitants** | **Time-to-peak of deaths for COVID-19** | **Cumulative hospital admissions at June 30** | **Cumulative hospital admissions at June 30 per 100000 inhabitants** | **Time-to-peak of hospital admissions for COVID-19** | **Cumulative ICU admissions at June 30** | **Cumulative ICU admissions at June 30 per 100000 inhabitants** | **Time-to-peak of ICU admissions for COVID-19** | **Percentage reduction of daily movements from Feb 10 to Feb 24** | **Percentage reduction of daily movements from Feb 10 to Mar 9** | **Percentage reduction of daily movements from Feb 10 to Mar 23** | **Total movements (per 1000)** |
| --- | --- | --- | --- | --- | --- | --- | --- | --- | --- | --- | --- | --- | --- | --- | --- | --- | --- |
| **Italy** | 60244639 | 235244 | 390 | 19 | 35033 | 58 | 20 | 86978 | 144 | 14 | 10344 | 17 | 15 | 14 | 55 | 82 | 3401085 |
| **AostaValley** | 125501 | 1187 | 946 | 17 | 145 | 116 | 20 | 332 | 265 | 16 | 47 | 37 | 14 | 19 | 43 | 87 | 3099 |
| Aosta | 125501 | 1187 | 946 | 17 | 145 | 116 | 20 | 332 | 265 | 16 | 47 | 37 | 14 | 19 | 43 | 87 | 3099 |
| **Piedmont** | 4341375 | 30856 | 711 | 32 | 4217 | 97 | 31 | 4783 | 110 | 34 | 1861 | 43 | 45 | 20 | 54 | 83 | 237174 |
| Verbano-Cusio-Ossola | 157455 | 1132 | 719 | 21 | 131 | 83 | 19 | 107 | 68 | 34 | 47 | 30 | 40 | 5 | 53 | 82 | 5479 |
| Biella | 174384 | 1017 | 583 | 22 | 197 | 113 | 21 | 56 | 32 | 13 | 4 | 2 | 37 | 8 | 52 | 80 | 4488 |
| Alessandria | 419037 | 4049 | 966 | 24 | 662 | 158 | 23 | 989 | 236 | 17 | 188 | 45 | 34 | 16 | 55 | 76 | 13950 |
| Asti | 213216 | 1866 | 875 | 41 | 266 | 125 | 44 | 235 | 110 | 48 | 26 | 12 | 48 | 12 | 52 | 76 | 11693 |
| Cuneo | 586568 | 2845 | 485 | 34 | 370 | 63 | 41 | 358 | 61 | 41 | 213 | 36 | 46 | 6 | 45 | 78 | 13517 |
| Novara | 368040 | 2779 | 755 | 30 | 379 | 103 | 25 | 820 | 223 | 46 | 210 | 57 | 49 | 16 | 57 | 82 | 15081 |
| Vercelli | 170296 | 1319 | 775 | 23 | 213 | 125 | 22 | 319 | 187 | 37 | 45 | 26 | 47 | 11 | 54 | 78 | 9316 |
| Turin | 2252379 | 15849 | 704 | 35 | 1999 | 89 | 34 | 1899 | 84 | 36 | 1128 | 50 | 44 | 23 | 54 | 85 | 163649 |
| **Liguria** | 1543127 | 9616 | 623 | 25 | 1555 | 101 | 25 | 4322 | 280 | 16 | 447 | 29 | 16 | 16 | 50 | 82 | 111123 |
| La Spezia | 219196 | 885 | 404 | 17 | 156 | 71 | 20 | 454 | 207 | 15 | 38 | 17 | 15 | 9 | 51 | 85 | 8746 |
| Genova | 835829 | 5611 | 671 | 28 | 936 | 112 | 26 | 2533 | 303 | 16 | 309 | 37 | 16 | 19 | 51 | 82 | 91306 |
| Savona | 274183 | 1550 | 565 | 32 | 231 | 84 | 25 | 697 | 254 | 17 | 59 | 22 | 15 | 8 | 46 | 82 | 6750 |
| Imperia | 213919 | 1570 | 734 | 21 | 232 | 108 | 29 | 638 | 298 | 16 | 41 | 19 | 18 | 5 | 44 | 81 | 4321 |
| **Lombardy** | 10103969 | 91772 | 908 | 18 | 16621 | 164 | 18 | 44631 | 442 | 14 | 4159 | 41 | 14 | 33 | 63 | 87 | 603882 |
| Monza-Brianza | 878267 | 5795 | 660 | 18 | 898 | 102 | 21 | 2660 | 303 | 15 | 225 | 26 | 15 | 31 | 60 | 87 | 51352 |
| Lodi | 230607 | 3571 | 1549 | 14 | 693 | 301 | 13 | 2148 | 931 | 7 | 241 | 105 | 4 | 48 | 57 | 77 | 11836 |
| Lecco | 337087 | 2839 | 842 | 16 | 470 | 139 | 17 | 1443 | 428 | 14 | 112 | 33 | 14 | 19 | 53 | 85 | 17840 |
| Mantua | 411062 | 3501 | 852 | 17 | 705 | 172 | 21 | 1237 | 301 | 14 | 92 | 22 | 14 | 19 | 52 | 78 | 13875 |
| Cremona | 358347 | 6504 | 1815 | 15 | 1118 | 312 | 16 | 3648 | 1018 | 11 | 342 | 95 | 11 | 36 | 56 | 79 | 13246 |
| Pavia | 546515 | 5575 | 1020 | 21 | 1309 | 240 | 21 | 2629 | 481 | 15 | 235 | 43 | 15 | 28 | 53 | 78 | 18549 |
| Brescia | 1268455 | 15473 | 1220 | 16 | 2679 | 211 | 17 | 8939 | 705 | 13 | 686 | 54 | 14 | 27 | 62 | 86 | 75132 |
| Bergamo | 1116384 | 14394 | 1289 | 14 | 3124 | 280 | 14 | 8522 | 763 | 12 | 912 | 82 | 13 | 29 | 65 | 87 | 51311 |
| Milan | 3279944 | 24499 | 747 | 24 | 4217 | 129 | 25 | 10443 | 318 | 15 | 981 | 30 | 15 | 38 | 67 | 89 | 291881 |
| Sondrio | 180941 | 1596 | 882 | 32 | 212 | 117 | 25 | 563 | 311 | 16 | 52 | 29 | 15 | 3 | 49 | 90 | 3954 |
| Como | 603828 | 4090 | 677 | 33 | 631 | 104 | 33 | 1273 | 211 | 16 | 146 | 24 | 17 | 20 | 55 | 86 | 23115 |
| Varese | 892532 | 3935 | 441 | 37 | 565 | 63 | 43 | 1126 | 126 | 18 | 135 | 15 | 17 | 24 | 60 | 86 | 31791 |
| **TrentinoSouthTyrol** | 1074819 | 7314 | 680 | 19 | 691 | 64 | 22 | 1661 | 155 | 15 | 235 | 22 | 15 | 7 | 52 | 86 | 33247 |
| Trento | 542739 | 4729 | 871 | 23 | 401 | 74 | 24 | 963 | 177 | 16 | 121 | 22 | 15 | 10 | 51 | 87 | 25103 |
| Bolzano | 532080 | 2585 | 486 | 17 | 290 | 55 | 20 | 698 | 131 | 15 | 114 | 21 | 15 | 4 | 55 | 86 | 8143 |
| Veneto | 4907704 | 18989 | 387 |  | 2022 | 41 |  | 5239 | 107 |  | 798 | 16 |  |  |  |  | 289620 |
| Rovigo | 233386 | 444 | 190 | 24 | 37 | 16 | 41 | 117 | 50 | 15 | 18 | 8 | 15 | 20 | 47 | 76 | 11220 |
| Padua | 939672 | 3947 | 420 | 15 | 311 | 33 | 30 | 887 | 94 | 15 | 136 | 14 | 14 | 32 | 56 | 82 | 59371 |
| Venezia | 851663 | 2685 | 315 | 17 | 299 | 35 | 39 | 794 | 93 | 16 | 144 | 17 | 13 | 25 | 58 | 85 | 68332 |
| Treviso | 888309 | 2708 | 305 | 17 | 328 | 37 | 21 | 980 | 110 | 14 | 137 | 15 | 14 | 19 | 54 | 84 | 27154 |
| Belluno | 201972 | 1166 | 577 | 23 | 112 | 55 | 44 | 210 | 104 | 17 | 16 | 8 | 15 | 16 | 59 | 94 | 5561 |
| Vicenza | 862363 | 2907 | 337 | 19 | 353 | 41 | 40 | 793 | 92 | 17 | 130 | 15 | 16 | 23 | 50 | 81 | 40976 |
| Verona | 930339 | 5132 | 552 | 20 | 582 | 63 | 32 | 1458 | 157 | 16 | 217 | 23 | 15 | 25 | 52 | 81 | 77006 |
| **FriuliVeneziaGiulia** | 1211357 | 3301 | 273 |  | 357 | 29 |  | 881 | 73 |  | 98 | 8 |  |  |  |  | 27695 |
| Friuli | 978081 | 1923 | 197 | 16 | 150 | 15 | 25 | 576 | 59 | 15 | 70 | 7 | 14 | 13 | 50 | 81 | 18194 |
| Trieste | 233276 | 1378 | 591 | 20 | 207 | 89 | 27 | 305 | 131 | 15 | 28 | 12 | 16 | 23 | 52 | 83 | 9501 |
| **EmiliaRomagna** | 4467118 | 27998 | 627 | 17 | 4373 | 98 | 19 | 10996 | 246 | 15 | 928 | 21 | 14 | 20 | 54 | 81 | 319331 |
| Rimini | 339796 | 2152 | 633 | 15 | 258 | 76 | 17 | 713 | 210 | 13 | 71 | 21 | 15 | 12 | 58 | 83 | 26381 |
| Forli-Cesena | 394833 | 1730 | 438 | 19 | 193 | 49 | 43 | 586 | 148 | 18 | 47 | 12 | 14 | 13 | 48 | 78 | 22326 |
| Ravenna | 389634 | 1037 | 266 | 16 | 84 | 22 | 27 | 400 | 103 | 16 | 30 | 8 | 15 | 10 | 43 | 76 | 19731 |
| Ferrara | 344840 | 1039 | 301 | 24 | 170 | 49 | 42 | 507 | 147 | 17 | 43 | 12 | 16 | 17 | 48 | 79 | 19982 |
| Bologna | 1017806 | 5242 | 515 | 21 | 736 | 72 | 35 | 2024 | 199 | 20 | 233 | 23 | 16 | 20 | 55 | 84 | 100333 |
| Modena | 707292 | 3845 | 544 | 16 | 472 | 67 | 19 | 1107 | 157 | 15 | 84 | 12 | 14 | 22 | 54 | 82 | 55252 |
| Reggio Emilia | 531751 | 4896 | 921 | 18 | 583 | 110 | 23 | 1535 | 289 | 15 | 60 | 11 | 11 | 22 | 54 | 81 | 31348 |
| Parma | 453930 | 3634 | 801 | 15 | 912 | 201 | 16 | 2171 | 478 | 13 | 98 | 22 | 11 | 23 | 58 | 78 | 34171 |
| Piacenza | 287236 | 4423 | 1540 | 16 | 965 | 336 | 16 | 1953 | 680 | 12 | 262 | 91 | 13 | 35 | 56 | 76 | 9807 |
| **Tuscany** | 3722729 | 9570 | 257 |  | 1076 | 29 |  | 2929 | 79 |  | 201 | 5 |  |  |  |  | 239717 |
| Prato | 258152 | 521 | 202 | 19 | 49 | 19 | 24 | 171 | 66 | 16 | 3 | 1 | 114 | 6 | 48 | 82 | 21535 |
| Grosseto | 220785 | 378 | 171 | 16 | 24 | 11 | 22 | 84 | 38 | 15 | 7 | 3 | 24 | 12 | 36 | 74 | 6436 |
| Siena | 266238 | 422 | 159 | 15 | 32 | 12 | 22 | 95 | 36 | 16 | 6 | 2 | 16 | 6 | 40 | 70 | 13232 |
| Arezzo | 341766 | 675 | 198 | 20 | 47 | 14 | 41 | 112 | 33 | 22 | 8 | 2 | 42 | 2 | 43 | 73 | 11650 |
| Pisa | 422310 | 901 | 213 | 17 | 90 | 21 | 21 | 280 | 66 | 16 | 12 | 3 | 33 | 1 | 47 | 79 | 20966 |
| Livorno | 333509 | 456 | 137 | 17 | 59 | 18 | 27 | 145 | 43 | 15 | 6 | 2 | 23 | 1 | 45 | 80 | 17075 |
| Firenze | 1004298 | 3118 | 310 | 24 | 390 | 39 | 40 | 1062 | 106 | 18 | 52 | 5 | 40 | 4 | 54 | 83 | 111222 |
| Pistoia | 293059 | 728 | 248 | 18 | 76 | 26 | 17 | 302 | 103 | 15 | 15 | 5 | 16 | 3 | 45 | 78 | 14048 |
| Lucca | 388678 | 1320 | 340 | 16 | 141 | 36 | 30 | 365 | 94 | 15 | 43 | 11 | 23 | 8 | 47 | 80 | 13726 |
| Massa-Carrara | 193934 | 1051 | 542 | 17 | 168 | 87 | 26 | 313 | 161 | 17 | 49 | 25 | 20 | 8 | 52 | 85 | 9826 |
| **Umbria** | 880285 | 1420 | 161 |  | 76 | 9 |  | 360 | 41 |  | 77 | 9 |  |  |  |  | 51405 |
| Terni | 224882 | 409 | 182 | 16 | 29 | 13 | 18 | 110 | 49 | 15 | 23 | 10 | 16 | 3 | 39 | 67 | 12186 |
| Perugia | 655403 | 1011 | 154 | 14 | 47 | 7 | 22 | 250 | 38 | 14 | 54 | 8 | 14 | 3 | 47 | 75 | 39219 |
| **Marche** | 1518400 | 6573 | 433 | 16 | 964 | 63 | 18 | 2121 | 140 | 14 | 265 | 17 | 13 | 3 | 49 | 79 | 61540 |
| Fermo | 173004 | 484 | 280 | 17 | 64 | 37 | 17 | 74 | 43 | 14 | 9 | 5 | 14 | 2 | 47 | 80 | 10601 |
| Ascoli Piceno | 206363 | 238 | 115 | 15 | 11 | 5 | 20 | 38 | 18 | 14 | 4 | 2 | 13 | 3 | 46 | 79 | 9473 |
| Macerata | 312146 | 1167 | 374 | 18 | 145 | 46 | 23 | 257 | 82 | 15 | 27 | 9 | 15 | 3 | 46 | 78 | 17007 |
| Ancona | 469750 | 1803 | 384 | 16 | 216 | 46 | 21 | 611 | 130 | 14 | 48 | 10 | 13 | 6 | 50 | 78 | 14851 |
| Pesaro | 357137 | 2881 | 807 | 15 | 528 | 148 | 17 | 1141 | 319 | 13 | 177 | 50 | 12 | 4 | 57 | 81 | 9607 |
| **Lazio** | 5865544 | 7771 | 132 |  | 867 | 15 |  | 2755 | 47 |  | 331 | 6 |  |  |  |  | 472390 |
| Frosinone | 485241 | 659 | 136 | 16 | 82 | 17 | 19 | 234 | 48 | 14 | 21 | 4 | 12 | 3 | 47 | 78 | 9935 |
| Latina | 576655 | 603 | 105 | 16 | 43 | 7 | 24 | 222 | 38 | 15 | 28 | 5 | 12 | 2 | 44 | 70 | 32560 |
| Roma | 4333274 | 5654 | 130 | 18 | 669 | 15 | 38 | 2161 | 50 | 17 | 262 | 6 | 15 | 4 | 56 | 84 | 406625 |
| Rieti | 154232 | 408 | 265 | 16 | 44 | 29 | 27 | 59 | 38 | 17 | 8 | 5 | 15 | 6 | 36 | 65 | 9225 |
| Viterbo | 316142 | 447 | 141 | 17 | 29 | 9 | 44 | 79 | 25 | 17 | 12 | 4 | 14 | 4 | 39 | 69 | 14045 |
| **Abruzzo** | 1305770 | 3256 | 249 |  | 458 | 35 |  | 1177 | 90 |  | 129 | 10 |  |  |  |  | 60741 |
| Chieti | 383189 | 818 | 213 | 19 | 121 | 32 | 35 | 306 | 80 | 19 | 36 | 9 | 15 | 2 | 50 | 77 | 16548 |
| Pescara | 318678 | 1578 | 495 | 18 | 254 | 80 | 35 | 569 | 179 | 16 | 66 | 21 | 14 | 4 | 56 | 83 | 27887 |
| Teramo | 307412 | 635 | 207 | 16 | 74 | 24 | 18 | 210 | 68 | 15 | 14 | 5 | 14 | 3 | 49 | 81 | 10229 |
| L'Aquila | 296491 | 225 | 76 | 16 | 9 | 3 | 18 | 92 | 31 | 13 | 13 | 4 | 17 | 20 | 48 | 87 | 6077 |
| **Molise** | 302265 | 427 | 141 |  | 21 | 7 |  | 99 | 33 |  | 10 | 3 |  |  |  |  | 12220 |
| Isernia | 83586 | 62 | 74 | 21 | 5 | 6 | 40 | 15 | 18 | 21 | 1 | 1 | 13 | 11 | 43 | 77 | 4249 |
| Campobasso | 218679 | 365 | 167 | 23 | 16 | 7 | 19 | 84 | 38 | 15 | 9 | 4 | 11 | 6 | 38 | 70 | 7972 |
| **Campania** | 5785861 | 4721 | 82 |  | 475 | 8 |  | 1055 | 18 |  | 123 | 2 |  |  |  |  | 316217 |
| Salerno | 1092779 | 696 | 64 | 17 | 64 | 6 | 20 | 168 | 15 | 15 | 15 | 1 | 15 | 6 | 53 | 81 | 46831 |
| Avellino | 413926 | 534 | 129 | 17 | 59 | 14 | 18 | 158 | 38 | 14 | 14 | 3 | 13 | 3 | 49 | 77 | 13898 |
| Napoli | 3082905 | 2620 | 85 | 18 | 293 | 10 | 24 | 557 | 18 | 16 | 89 | 3 | 16 | 7 | 56 | 83 | 209547 |
| Benevento | 274080 | 208 | 76 | 19 | 16 | 6 | 33 | 54 | 20 | 15 | 2 | 1 | 12 | 1 | 45 | 75 | 10934 |
| Caserta | 922171 | 663 | 72 | 18 | 43 | 5 | 18 | 118 | 13 | 16 | 3 | 0 | 14 | 3 | 50 | 80 | 35006 |
| **Apulia** | 4008296 | 4496 | 112 |  | 546 | 14 |  | 1699 | 42 |  | 270 | 7 |  |  |  |  | 150610 |
| BAT | 388390 | 380 | 98 | 23 | 63 | 16 | 44 | 158 | 41 | 33 | 31 | 8 | 37 | 5 | 46 | 73 | 13410 |
| Lecce | 791122 | 521 | 66 | 16 | 76 | 10 | 19 | 234 | 30 | 15 | 26 | 3 | 19 | 8 | 57 | 83 | 5008 |
| Brindisi | 390456 | 658 | 169 | 23 | 66 | 17 | 36 | 180 | 46 | 19 | 15 | 4 | 16 | 4 | 49 | 78 | 12063 |
| Taranto | 572772 | 281 | 49 | 18 | 33 | 6 | 43 | 131 | 23 | 16 | 19 | 3 | 21 | 6 | 47 | 75 | 29660 |
| Bari | 1249246 | 1488 | 119 | 20 | 152 | 12 | 27 | 535 | 43 | 16 | 96 | 8 | 16 | 1 | 54 | 82 | 71351 |
| Foggia | 616310 | 1168 | 190 | 24 | 156 | 25 | 31 | 461 | 75 | 18 | 83 | 13 | 17 | 2 | 40 | 67 | 19117 |
| Basilicata | 556934 | 366 | 66 |  | 30 | 5 |  | 120 | 22 |  | 30 | 5 |  |  |  |  | 18617 |
| Matera | 195998 | 193 | 98 | 19 | 9 | 5 | 45 | 58 | 30 | 24 | 13 | 7 | 16 | 7 | 40 | 66 | 8829 |
| Potenza | 360936 | 173 | 48 | 15 | 21 | 6 | 23 | 62 | 17 | 16 | 17 | 5 | 14 | 2 | 40 | 71 | 9788 |
| **Calabria** | 1924701 | 1193 | 62 |  | 97 | 5 |  | 356 | 18 |  | 50 | 3 |  |  |  |  | 41853 |
| Vibo Valentia | 157469 | 85 | 54 | 15 | 5 | 3 | 18 | 22 | 14 | 16 | 5 | 3 | 14 | 3 | 36 | 60 | 4922 |
| Crotone | 170718 | 118 | 69 | 14 | 7 | 4 | 16 | 34 | 20 | 14 | 8 | 5 | 14 | 2 | 47 | 75 | 3036 |
| Reggio Calabria | 541278 | 315 | 58 | 16 | 19 | 4 | 16 | 96 | 18 | 15 | 13 | 2 | 15 | 3 | 46 | 70 | 20736 |
| Catanzaro | 354851 | 204 | 57 | 17 | 32 | 9 | 30 | 100 | 28 | 18 | 11 | 3 | 18 | 2 | 49 | 74 | 6184 |
| Cosenza | 700385 | 471 | 67 | 23 | 34 | 5 | 32 | 104 | 15 | 16 | 13 | 2 | 15 | 5 | 50 | 81 | 6975 |
| **Sicily** | 4968410 | 3054 | 61 |  | 309 | 6 |  | 1132 | 23 |  | 201 | 4 |  |  |  |  | 268873 |
| Siracusa | 397037 | 334 | 84 | 22 | 45 | 11 | 44 | 165 | 42 | 20 | 26 | 7 | 25 | 5 | 45 | 75 | 21293 |
| Ragusa | 321215 | 91 | 28 | 20 | 6 | 2 | 20 | 26 | 8 | 16 | 5 | 2 | 16 | 6 | 34 | 60 | 6805 |
| Catania | 1104974 | 777 | 70 | 17 | 97 | 9 | 28 | 336 | 30 | 17 | 84 | 8 | 16 | 2 | 54 | 81 | 95876 |
| Enna | 162368 | 431 | 265 | 16 | 35 | 22 | 22 | 126 | 78 | 15 | 15 | 9 | 15 | 7 | 38 | 70 | 7241 |
| Caltanissetta | 260779 | 187 | 72 | 19 | 13 | 5 | 21 | 55 | 21 | 16 | 11 | 4 | 14 | 3 | 42 | 70 | 8861 |
| Agrigento | 429611 | 137 | 32 | 16 | 13 | 3 | 19 | 38 | 9 | 15 | 4 | 1 | 15 | 7 | 47 | 76 | 7223 |
| Messina | 620721 | 459 | 74 | 16 | 57 | 9 | 29 | 206 | 33 | 16 | 19 | 3 | 16 | 0 | 48 | 75 | 27016 |
| Palermo | 1243328 | 499 | 40 | 18 | 37 | 3 | 30 | 140 | 11 | 16 | 24 | 2 | 14 | 10 | 53 | 83 | 89215 |
| Trapani | 428377 | 139 | 32 | 16 | 6 | 1 | 23 | 40 | 9 | 15 | 13 | 3 | 14 | 2 | 49 | 76 | 5341 |
| **Sardinia** | 1630474 | 1364 | 84 |  | 133 | 8 |  | 330 | 20 |  | 84 | 5 |  |  |  |  | 81732 |
| Sud Sardegna | 347005 | 119 | 34 | 16 | 16 | 5 | 33 | 40 | 12 | 17 | 9 | 3 | 46 | 3 | 45 | 75 | 11713 |
| Oristano | 156078 | 69 | 44 | 24 | 5 | 3 | 42 | 21 | 13 | 16 | 5 | 3 | 17 | 28 | 44 | 72 | 3910 |
| Cagliari | 430914 | 231 | 54 | 20 | 16 | 4 | 35 | 51 | 12 | 16 | 9 | 2 | 14 | 3 | 54 | 81 | 38768 |
| Nuoro | 206843 | 94 | 45 | 15 | 10 | 5 | 20 | 27 | 13 | 16 | 6 | 3 | 15 | 7 | 42 | 71 | 4929 |
| Sassari | 489634 | 851 | 174 | 17 | 86 | 18 | 23 | 191 | 39 | 16 | 55 | 11 | 19 | 3 | 48 | 75 | 22413 |

**Supplementary Table 2.** Summary of population size, cumulative number of COVID-19 outcomes by June 30, 2020, time-to-peak of health endpoints, mobile phone movements reductions at different time points according to reasons for travel.

| **Province** | **Population** | **Cumulative cases at June 30** | **Cumulative cases at June 30 per 100000 inhabitants** | **Time-to-peak of Sars-Cov-2 infections** | **Cumulative deaths at June 30** | **Cumulative deaths at June 30 per 100000 inhabitants** | **Time-to-peak of deaths for COVID-19** | **Cumulative hospital admissions at June 30** | **Cumulative hospital admissions at June 30 per 100000 inhabitants** | **Time-to-peak of hospital admissions for COVID-19** | **Cumulative ICU admissions at June 30** | **Cumulative ICU admissions at June 30 per 100000 inhabitants** | **Time-to-peak of ICU admissions for COVID-19** | **Percentage reduction of daily movements unrelated to work from Feb 10 to Feb 24** | **Percentage reduction of daily movements for work from Feb 10 to Feb 24** | **Percentage reduction of daily movements unrelated to work from Feb 10 to Mar 9** | **Percentage reduction of daily movements for work from Feb 10 to Mar 9** | **Percentage reduction of daily movements unrelated to work from Feb 10 to Mar 23** | **Percentage reduction of daily movements for work from Feb 10 to Mar 23** |
| --- | --- | --- | --- | --- | --- | --- | --- | --- | --- | --- | --- | --- | --- | --- | --- | --- | --- | --- | --- |
| **Italy** | 60244639 | 235244 | 390 | 19 | 35033 | 58 | 20 | 86978 | 144 | 14 | 10344 | 17 | 15 | 10 | 20 | 58 | 49 | 84 | 79 |
| **AostaValley** | 125501 | 1187 | 946 | 17 | 145 | 116 | 20 | 332 | 265 | 16 | 47 | 37 | 14 | 23 | 7 | 43 | 43 | 88 | 85 |
| Aosta | 125501 | 1187 | 946 | 17 | 145 | 116 | 20 | 332 | 265 | 16 | 47 | 37 | 14 | 23 | 7 | 43 | 43 | 88 | 85 |
| **Piedmont** | 4341375 | 30856 | 711 | 32 | 4217 | 97 | 31 | 4783 | 110 | 34 | 1861 | 43 | 45 | 15 | 28 | 58 | 47 | 85 | 80 |
| Verbano-Cusio-Ossola | 157455 | 1132 | 719 | 21 | 131 | 83 | 19 | 107 | 68 | 34 | 47 | 30 | 40 | 1 | 23 | 56 | 40 | 83 | 79 |
| Biella | 174384 | 1017 | 583 | 22 | 197 | 113 | 21 | 56 | 32 | 13 | 4 | 2 | 37 | 4 | 19 | 57 | 39 | 82 | 75 |
| Alessandria | 419037 | 4049 | 966 | 24 | 662 | 158 | 23 | 989 | 236 | 17 | 188 | 45 | 34 | 12 | 25 | 57 | 48 | 76 | 74 |
| Asti | 213216 | 1866 | 875 | 41 | 266 | 125 | 44 | 235 | 110 | 48 | 26 | 12 | 48 | 8 | 22 | 56 | 42 | 77 | 72 |
| Cuneo | 586568 | 2845 | 485 | 34 | 370 | 63 | 41 | 358 | 61 | 41 | 213 | 36 | 46 | 1 | 22 | 48 | 36 | 79 | 74 |
| Novara | 368040 | 2779 | 755 | 30 | 379 | 103 | 25 | 820 | 223 | 46 | 210 | 57 | 49 | 13 | 23 | 62 | 45 | 84 | 76 |
| Vercelli | 170296 | 1319 | 775 | 23 | 213 | 125 | 22 | 319 | 187 | 37 | 45 | 26 | 47 | 7 | 22 | 58 | 43 | 80 | 73 |
| Turin | 2252379 | 15849 | 704 | 35 | 1999 | 89 | 34 | 1899 | 84 | 36 | 1128 | 50 | 44 | 18 | 29 | 59 | 48 | 88 | 81 |
| **Liguria** | 1543127 | 9616 | 623 | 25 | 1555 | 101 | 25 | 4322 | 280 | 16 | 447 | 29 | 16 | 12 | 20 | 55 | 45 | 86 | 78 |
| La Spezia | 219196 | 885 | 404 | 17 | 156 | 71 | 20 | 454 | 207 | 15 | 38 | 17 | 15 | 6 | 15 | 56 | 39 | 87 | 80 |
| Genova | 835829 | 5611 | 671 | 28 | 936 | 112 | 26 | 2533 | 303 | 16 | 309 | 37 | 16 | 17 | 21 | 56 | 46 | 87 | 78 |
| Savona | 274183 | 1550 | 565 | 32 | 231 | 84 | 25 | 697 | 254 | 17 | 59 | 22 | 15 | 16 | 16 | 48 | 41 | 83 | 77 |
| Imperia | 213919 | 1570 | 734 | 21 | 232 | 108 | 29 | 638 | 298 | 16 | 41 | 19 | 18 | 8 | 13 | 44 | 44 | 81 | 80 |
| **Lombardy** | 10103969 | 91772 | 908 | 18 | 16621 | 164 | 18 | 44631 | 442 | 14 | 4159 | 41 | 14 | 29 | 37 | 67 | 59 | 88 | 84 |
| Monza-Brianza | 878267 | 5795 | 660 | 18 | 898 | 102 | 21 | 2660 | 303 | 15 | 225 | 26 | 15 | 28 | 35 | 64 | 53 | 89 | 83 |
| Lodi | 230607 | 3571 | 1549 | 14 | 693 | 301 | 13 | 2148 | 931 | 7 | 241 | 105 | 4 | 47 | 52 | 61 | 50 | 78 | 74 |
| Lecco | 337087 | 2839 | 842 | 16 | 470 | 139 | 17 | 1443 | 428 | 14 | 112 | 33 | 14 | 14 | 28 | 58 | 44 | 87 | 80 |
| Mantua | 411062 | 3501 | 852 | 17 | 705 | 172 | 21 | 1237 | 301 | 14 | 92 | 22 | 14 | 16 | 25 | 56 | 40 | 80 | 75 |
| Cremona | 358347 | 6504 | 1815 | 15 | 1118 | 312 | 16 | 3648 | 1018 | 11 | 342 | 95 | 11 | 34 | 38 | 61 | 45 | 81 | 74 |
| Pavia | 546515 | 5575 | 1020 | 21 | 1309 | 240 | 21 | 2629 | 481 | 15 | 235 | 43 | 15 | 25 | 33 | 57 | 45 | 80 | 74 |
| Brescia | 1268455 | 15473 | 1220 | 16 | 2679 | 211 | 17 | 8939 | 705 | 13 | 686 | 54 | 14 | 22 | 34 | 66 | 55 | 88 | 83 |
| Bergamo | 1116384 | 14394 | 1289 | 14 | 3124 | 280 | 14 | 8522 | 763 | 12 | 912 | 82 | 13 | 24 | 38 | 68 | 59 | 89 | 85 |
| Milan | 3279944 | 24499 | 747 | 24 | 4217 | 129 | 25 | 10443 | 318 | 15 | 981 | 30 | 15 | 36 | 40 | 70 | 64 | 90 | 87 |
| Sondrio | 180941 | 1596 | 882 | 32 | 212 | 117 | 25 | 563 | 311 | 16 | 52 | 29 | 15 | 10 | 25 | 51 | 42 | 92 | 81 |
| Como | 603828 | 4090 | 677 | 33 | 631 | 104 | 33 | 1273 | 211 | 16 | 146 | 24 | 17 | 16 | 27 | 60 | 45 | 88 | 81 |
| Varese | 892532 | 3935 | 441 | 37 | 565 | 63 | 43 | 1126 | 126 | 18 | 135 | 15 | 17 | 21 | 30 | 65 | 50 | 89 | 81 |
| **TrentinoSouthTyrol** | 1074819 | 7314 | 680 | 19 | 691 | 64 | 22 | 1661 | 155 | 15 | 235 | 22 | 15 | 1 | 26 | 56 | 43 | 89 | 79 |
| Trento | 542739 | 4729 | 871 | 23 | 401 | 74 | 24 | 963 | 177 | 16 | 121 | 22 | 15 | 1 | 28 | 55 | 43 | 91 | 78 |
| Bolzano | 532080 | 2585 | 486 | 17 | 290 | 55 | 20 | 698 | 131 | 15 | 114 | 21 | 15 | 5 | 9 | 57 | 42 | 86 | 82 |
| Veneto | 4907704 | 18989 | 387 | 18 | 2022 | 41 | 34 | 5239 | 107 | 15 | 798 | 16 | 14 | 20 | 32 | 58 | 47 | 85 | 78 |
| Rovigo | 233386 | 444 | 190 | 24 | 37 | 16 | 41 | 117 | 50 | 15 | 18 | 8 | 15 | 17 | 28 | 51 | 37 | 78 | 69 |
| Padua | 939672 | 3947 | 420 | 15 | 311 | 33 | 30 | 887 | 94 | 15 | 136 | 14 | 14 | 28 | 39 | 61 | 47 | 85 | 76 |
| Venezia | 851663 | 2685 | 315 | 17 | 299 | 35 | 39 | 794 | 93 | 16 | 144 | 17 | 13 | 22 | 29 | 63 | 52 | 87 | 82 |
| Treviso | 888309 | 2708 | 305 | 17 | 328 | 37 | 21 | 980 | 110 | 14 | 137 | 15 | 14 | 15 | 27 | 61 | 40 | 86 | 78 |
| Belluno | 201972 | 1166 | 577 | 23 | 112 | 55 | 44 | 210 | 104 | 17 | 16 | 8 | 15 | 21 | 11 | 63 | 44 | 95 | 85 |
| Vicenza | 862363 | 2907 | 337 | 19 | 353 | 41 | 40 | 793 | 92 | 17 | 130 | 15 | 16 | 18 | 33 | 55 | 41 | 84 | 76 |
| Verona | 930339 | 5132 | 552 | 20 | 582 | 63 | 32 | 1458 | 157 | 16 | 217 | 23 | 15 | 19 | 34 | 55 | 48 | 83 | 78 |
| **FriuliVeneziaGiulia** | 1211357 | 3301 | 273 | 17 | 357 | 29 | 26 | 881 | 73 | 15 | 98 | 8 | 15 | 12 | 28 | 55 | 40 | 84 | 77 |
| Friuli | 978081 | 1923 | 197 | 16 | 150 | 15 | 25 | 576 | 59 | 15 | 70 | 7 | 14 | 10 | 24 | 54 | 37 | 82 | 77 |
| Trieste | 233276 | 1378 | 591 | 20 | 207 | 89 | 27 | 305 | 131 | 15 | 28 | 12 | 16 | 17 | 33 | 55 | 45 | 86 | 77 |
| **EmiliaRomagna** | 4467118 | 27998 | 627 | 17 | 4373 | 98 | 19 | 10996 | 246 | 15 | 928 | 21 | 14 | 15 | 27 | 58 | 46 | 84 | 77 |
| Rimini | 339796 | 2152 | 633 | 15 | 258 | 76 | 17 | 713 | 210 | 13 | 71 | 21 | 15 | 7 | 23 | 62 | 48 | 85 | 79 |
| Forli-Cesena | 394833 | 1730 | 438 | 19 | 193 | 49 | 43 | 586 | 148 | 18 | 47 | 12 | 14 | 8 | 22 | 54 | 37 | 81 | 72 |
| Ravenna | 389634 | 1037 | 266 | 16 | 84 | 22 | 27 | 400 | 103 | 16 | 30 | 8 | 15 | 5 | 20 | 48 | 33 | 79 | 71 |
| Ferrara | 344840 | 1039 | 301 | 24 | 170 | 49 | 42 | 507 | 147 | 17 | 43 | 12 | 16 | 11 | 28 | 52 | 40 | 82 | 73 |
| Bologna | 1017806 | 5242 | 515 | 21 | 736 | 72 | 35 | 2024 | 199 | 20 | 233 | 23 | 16 | 16 | 26 | 59 | 50 | 86 | 80 |
| Modena | 707292 | 3845 | 544 | 16 | 472 | 67 | 19 | 1107 | 157 | 15 | 84 | 12 | 14 | 19 | 28 | 59 | 46 | 84 | 78 |
| Reggio Emilia | 531751 | 4896 | 921 | 18 | 583 | 110 | 23 | 1535 | 289 | 15 | 60 | 11 | 11 | 18 | 29 | 59 | 45 | 83 | 78 |
| Parma | 453930 | 3634 | 801 | 15 | 912 | 201 | 16 | 2171 | 478 | 13 | 98 | 22 | 11 | 19 | 31 | 63 | 50 | 81 | 73 |
| Piacenza | 287236 | 4423 | 1540 | 16 | 965 | 336 | 16 | 1953 | 680 | 12 | 262 | 91 | 13 | 32 | 43 | 58 | 51 | 76 | 74 |
| **Tuscany** | 3722729 | 9570 | 257 | 18 | 1076 | 29 | 31 | 2929 | 79 | 16 | 201 | 5 | 24 | 0 | 5 | 53 | 44 | 82 | 77 |
| Prato | 258152 | 521 | 202 | 19 | 49 | 19 | 24 | 171 | 66 | 16 | 3 | 1 | 114 | 6 | 6 | 53 | 41 | 84 | 79 |
| Grosseto | 220785 | 378 | 171 | 16 | 24 | 11 | 22 | 84 | 38 | 15 | 7 | 3 | 24 | 14 | 0 | 39 | 26 | 76 | 68 |
| Siena | 266238 | 422 | 159 | 15 | 32 | 12 | 22 | 95 | 36 | 16 | 6 | 2 | 16 | 8 | 2 | 44 | 30 | 71 | 66 |
| Arezzo | 341766 | 675 | 198 | 20 | 47 | 14 | 41 | 112 | 33 | 22 | 8 | 2 | 42 | 4 | 3 | 45 | 37 | 73 | 72 |
| Pisa | 422310 | 901 | 213 | 17 | 90 | 21 | 21 | 280 | 66 | 16 | 12 | 3 | 33 | 0 | 3 | 51 | 37 | 81 | 73 |
| Livorno | 333509 | 456 | 137 | 17 | 59 | 18 | 27 | 145 | 43 | 15 | 6 | 2 | 23 | 1 | 5 | 48 | 37 | 83 | 73 |
| Firenze | 1004298 | 3118 | 310 | 24 | 390 | 39 | 40 | 1062 | 106 | 18 | 52 | 5 | 40 | 3 | 5 | 57 | 49 | 85 | 80 |
| Pistoia | 293059 | 728 | 248 | 18 | 76 | 26 | 17 | 302 | 103 | 15 | 15 | 5 | 16 | 1 | 6 | 49 | 38 | 80 | 73 |
| Lucca | 388678 | 1320 | 340 | 16 | 141 | 36 | 30 | 365 | 94 | 15 | 43 | 11 | 23 | 14 | 3 | 50 | 39 | 82 | 75 |
| Massa-Carrara | 193934 | 1051 | 542 | 17 | 168 | 87 | 26 | 313 | 161 | 17 | 49 | 25 | 20 | 6 | 13 | 58 | 41 | 88 | 80 |
| **Umbria** | 880285 | 1420 | 161 | 15 | 76 | 9 | 20 | 360 | 41 | 15 | 77 | 9 | 15 | 4 | 1 | 48 | 38 | 75 | 70 |
| Terni | 224882 | 409 | 182 | 16 | 29 | 13 | 18 | 110 | 49 | 15 | 23 | 10 | 16 | 5 | 0 | 41 | 32 | 69 | 63 |
| Perugia | 655403 | 1011 | 154 | 14 | 47 | 7 | 22 | 250 | 38 | 14 | 54 | 8 | 14 | 4 | 1 | 50 | 39 | 77 | 71 |
| **Marche** | 1518400 | 6573 | 433 | 16 | 964 | 63 | 18 | 2121 | 140 | 14 | 265 | 17 | 13 | 1 | 14 | 52 | 39 | 80 | 76 |
| Fermo | 173004 | 484 | 280 | 17 | 64 | 37 | 17 | 74 | 43 | 14 | 9 | 5 | 14 | 2 | 15 | 51 | 37 | 81 | 77 |
| Ascoli Piceno | 206363 | 238 | 115 | 15 | 11 | 5 | 20 | 38 | 18 | 14 | 4 | 2 | 13 | 8 | 12 | 50 | 34 | 81 | 73 |
| Macerata | 312146 | 1167 | 374 | 18 | 145 | 46 | 23 | 257 | 82 | 15 | 27 | 9 | 15 | 0 | 14 | 49 | 36 | 79 | 75 |
| Ancona | 469750 | 1803 | 384 | 16 | 216 | 46 | 21 | 611 | 130 | 14 | 48 | 10 | 13 | 3 | 14 | 54 | 39 | 79 | 75 |
| Pesaro | 357137 | 2881 | 807 | 15 | 528 | 148 | 17 | 1141 | 319 | 13 | 177 | 50 | 12 | 0 | 16 | 61 | 47 | 82 | 80 |
| **Lazio** | 5865544 | 7771 | 132 | 17 | 867 | 15 | 35 | 2755 | 47 | 16 | 331 | 6 | 14 | 2 | 4 | 57 | 52 | 84 | 80 |
| Frosinone | 485241 | 659 | 136 | 16 | 82 | 17 | 19 | 234 | 48 | 14 | 21 | 4 | 12 | 5 | 2 | 49 | 42 | 79 | 75 |
| Latina | 576655 | 603 | 105 | 16 | 43 | 7 | 24 | 222 | 38 | 15 | 28 | 5 | 12 | 6 | 5 | 45 | 40 | 72 | 67 |
| Roma | 4333274 | 5654 | 130 | 18 | 669 | 15 | 38 | 2161 | 50 | 17 | 262 | 6 | 15 | 3 | 4 | 59 | 53 | 86 | 81 |
| Rieti | 154232 | 408 | 265 | 16 | 44 | 29 | 27 | 59 | 38 | 17 | 8 | 5 | 15 | 8 | 1 | 36 | 33 | 65 | 65 |
| Viterbo | 316142 | 447 | 141 | 17 | 29 | 9 | 44 | 79 | 25 | 17 | 12 | 4 | 14 | 6 | 2 | 40 | 36 | 69 | 67 |
| **Abruzzo** | 1305770 | 3256 | 249 | 17 | 458 | 35 | 28 | 1177 | 90 | 16 | 129 | 10 | 15 | 5 | 11 | 55 | 47 | 83 | 78 |
| Chieti | 383189 | 818 | 213 | 19 | 121 | 32 | 35 | 306 | 80 | 19 | 36 | 9 | 15 | 2 | 11 | 52 | 44 | 78 | 74 |
| Pescara | 318678 | 1578 | 495 | 18 | 254 | 80 | 35 | 569 | 179 | 16 | 66 | 21 | 14 | 1 | 13 | 59 | 52 | 85 | 79 |
| Teramo | 307412 | 635 | 207 | 16 | 74 | 24 | 18 | 210 | 68 | 15 | 14 | 5 | 14 | 8 | 11 | 51 | 41 | 83 | 77 |
| L'Aquila | 296491 | 225 | 76 | 16 | 9 | 3 | 18 | 92 | 31 | 13 | 13 | 4 | 17 | 26 | 1 | 50 | 43 | 89 | 81 |
| **Molise** | 302265 | 427 | 141 | 22 | 21 | 7 | 21 | 99 | 33 | 15 | 10 | 3 | 12 | 11 | 3 | 42 | 35 | 73 | 71 |
| Isernia | 83586 | 62 | 74 | 21 | 5 | 6 | 40 | 15 | 18 | 21 | 1 | 1 | 13 | 15 | 1 | 45 | 38 | 78 | 73 |
| Campobasso | 218679 | 365 | 167 | 23 | 16 | 7 | 19 | 84 | 38 | 15 | 9 | 4 | 11 | 9 | 5 | 40 | 34 | 70 | 70 |
| **Campania** | 5785861 | 4721 | 82 | 18 | 475 | 8 | 22 | 1055 | 18 | 15 | 123 | 2 | 15 | 0 | 15 | 59 | 48 | 85 | 78 |
| Salerno | 1092779 | 696 | 64 | 17 | 64 | 6 | 20 | 168 | 15 | 15 | 15 | 1 | 15 | 2 | 19 | 57 | 47 | 83 | 76 |
| Avellino | 413926 | 534 | 129 | 17 | 59 | 14 | 18 | 158 | 38 | 14 | 14 | 3 | 13 | 2 | 13 | 52 | 41 | 78 | 75 |
| Napoli | 3082905 | 2620 | 85 | 18 | 293 | 10 | 24 | 557 | 18 | 16 | 89 | 3 | 16 | 1 | 15 | 61 | 50 | 87 | 79 |
| Benevento | 274080 | 208 | 76 | 19 | 16 | 6 | 33 | 54 | 20 | 15 | 2 | 1 | 12 | 3 | 11 | 48 | 37 | 76 | 72 |
| Caserta | 922171 | 663 | 72 | 18 | 43 | 5 | 18 | 118 | 13 | 16 | 3 | 0 | 14 | 2 | 13 | 55 | 41 | 83 | 75 |
| **Apulia** | 4008296 | 4496 | 112 | 20 | 546 | 14 | 32 | 1699 | 42 | 17 | 270 | 7 | 18 | 3 | 8 | 54 | 42 | 80 | 73 |
| BAT | 388390 | 380 | 98 | 23 | 63 | 16 | 44 | 158 | 41 | 33 | 31 | 8 | 37 | 9 | 3 | 50 | 39 | 75 | 69 |
| Lecce | 791122 | 521 | 66 | 16 | 76 | 10 | 19 | 234 | 30 | 15 | 26 | 3 | 19 | 12 | 5 | 62 | 44 | 88 | 72 |
| Brindisi | 390456 | 658 | 169 | 23 | 66 | 17 | 36 | 180 | 46 | 19 | 15 | 4 | 16 | 9 | 7 | 54 | 39 | 81 | 69 |
| Taranto | 572772 | 281 | 49 | 18 | 33 | 6 | 43 | 131 | 23 | 16 | 19 | 3 | 21 | 0 | 16 | 52 | 38 | 78 | 68 |
| Bari | 1249246 | 1488 | 119 | 20 | 152 | 12 | 27 | 535 | 43 | 16 | 96 | 8 | 16 | 1 | 6 | 59 | 45 | 85 | 76 |
| Foggia | 616310 | 1168 | 190 | 24 | 156 | 25 | 31 | 461 | 75 | 18 | 83 | 13 | 17 | 6 | 7 | 42 | 35 | 67 | 67 |
| Basilicata | 556934 | 366 | 66 | 17 | 30 | 5 | 29 | 120 | 22 | 18 | 30 | 5 | 15 | 7 | 5 | 43 | 31 | 69 | 67 |
| Matera | 195998 | 193 | 98 | 19 | 9 | 5 | 45 | 58 | 30 | 24 | 13 | 7 | 16 | 12 | 5 | 43 | 31 | 67 | 66 |
| Potenza | 360936 | 173 | 48 | 15 | 21 | 6 | 23 | 62 | 17 | 16 | 17 | 5 | 14 | 4 | 5 | 43 | 31 | 72 | 69 |
| **Calabria** | 1924701 | 1193 | 62 | 17 | 97 | 5 | 23 | 356 | 18 | 16 | 50 | 3 | 15 | 0 | 6 | 47 | 43 | 73 | 69 |
| Vibo Valentia | 157469 | 85 | 54 | 15 | 5 | 3 | 18 | 22 | 14 | 16 | 5 | 3 | 14 | 3 | 3 | 38 | 29 | 61 | 56 |
| Crotone | 170718 | 118 | 69 | 14 | 7 | 4 | 16 | 34 | 20 | 14 | 8 | 5 | 14 | 3 | 0 | 50 | 38 | 77 | 68 |
| Reggio Calabria | 541278 | 315 | 58 | 16 | 19 | 4 | 16 | 96 | 18 | 15 | 13 | 2 | 15 | 2 | 7 | 46 | 46 | 70 | 70 |
| Catanzaro | 354851 | 204 | 57 | 17 | 32 | 9 | 30 | 100 | 28 | 18 | 11 | 3 | 18 | 0 | 7 | 52 | 39 | 77 | 67 |
| Cosenza | 700385 | 471 | 67 | 23 | 34 | 5 | 32 | 104 | 15 | 16 | 13 | 2 | 15 | 8 | 7 | 53 | 42 | 83 | 74 |
| **Sicily** | 4968410 | 3054 | 61 | 17 | 309 | 6 | 28 | 1132 | 23 | 16 | 201 | 4 | 16 | 1 | 10 | 54 | 46 | 82 | 75 |
| Siracusa | 397037 | 334 | 84 | 22 | 45 | 11 | 44 | 165 | 42 | 20 | 26 | 7 | 25 | 11 | 8 | 48 | 40 | 77 | 71 |
| Ragusa | 321215 | 91 | 28 | 20 | 6 | 2 | 20 | 26 | 8 | 16 | 5 | 2 | 16 | 9 | 4 | 37 | 24 | 63 | 50 |
| Catania | 1104974 | 777 | 70 | 17 | 97 | 9 | 28 | 336 | 30 | 17 | 84 | 8 | 16 | 2 | 3 | 58 | 48 | 85 | 76 |
| Enna | 162368 | 431 | 265 | 16 | 35 | 22 | 22 | 126 | 78 | 15 | 15 | 9 | 15 | 10 | 1 | 39 | 36 | 70 | 69 |
| Caltanissetta | 260779 | 187 | 72 | 19 | 13 | 5 | 21 | 55 | 21 | 16 | 11 | 4 | 14 | 6 | 5 | 45 | 34 | 73 | 62 |
| Agrigento | 429611 | 137 | 32 | 16 | 13 | 3 | 19 | 38 | 9 | 15 | 4 | 1 | 15 | 13 | 10 | 50 | 41 | 78 | 71 |
| Messina | 620721 | 459 | 74 | 16 | 57 | 9 | 29 | 206 | 33 | 16 | 19 | 3 | 16 | 5 | 8 | 50 | 46 | 76 | 73 |
| Palermo | 1243328 | 499 | 40 | 18 | 37 | 3 | 30 | 140 | 11 | 16 | 24 | 2 | 14 | 3 | 19 | 58 | 47 | 87 | 78 |
| Trapani | 428377 | 139 | 32 | 16 | 6 | 1 | 23 | 40 | 9 | 15 | 13 | 3 | 14 | 7 | 10 | 51 | 45 | 77 | 74 |
| **Sardinia** | 1630474 | 1364 | 84 | 17 | 133 | 8 | 25 | 330 | 20 | 16 | 84 | 5 | 18 | 5 | 9 | 53 | 45 | 81 | 71 |
| Sud Sardegna | 347005 | 119 | 34 | 16 | 16 | 5 | 33 | 40 | 12 | 17 | 9 | 3 | 46 | 8 | 5 | 49 | 37 | 80 | 65 |
| Oristano | 156078 | 69 | 44 | 24 | 5 | 3 | 42 | 21 | 13 | 16 | 5 | 3 | 17 | 33 | 15 | 47 | 34 | 75 | 61 |
| Cagliari | 430914 | 231 | 54 | 20 | 16 | 4 | 35 | 51 | 12 | 16 | 9 | 2 | 14 | 1 | 9 | 58 | 48 | 84 | 74 |
| Nuoro | 206843 | 94 | 45 | 15 | 10 | 5 | 20 | 27 | 13 | 16 | 6 | 3 | 15 | 11 | 3 | 47 | 30 | 74 | 62 |
| Sassari | 489634 | 851 | 174 | 17 | 86 | 18 | 23 | 191 | 39 | 16 | 55 | 11 | 19 | 3 | 12 | 49 | 45 | 78 | 71 |

**Supplementary Table 3.** Summary of population size, cumulative number of COVID-19 outcomes by June 30, 2020, time-to-peak of health endpoints, mobile phone movements reductions at different time points according to means of transportation.

| **Province** | **Population** | **Cumulative cases at June 30** | **Cumulative cases at June 30 per 100000 inhabitants** | **Time-to-peak of Sars-Cov-2 infections** | **Cumulative deaths at June 30** | **Cumulative deaths at June 30 per 100000 inhabitants** | **Time-to-peak of deaths for COVID-19** | **Cumulative hospital admissions at June 30** | **Cumulative hospital admissions at June 30 per 100000 inhabitants** | **Time-to-peak of hospital admissions for COVID-19** | **Cumulative ICU admissions at June 30** | **Cumulative ICU admissions at June 30 per 100000 inhabitants** | **Time-to-peak of ICU admissions for COVID-19** | **Percentage reduction of daily movements (unclassified) from Feb 10 to Feb 24** | **Percentage reduction of daily movements on plane from Feb 10 to Feb 24** | **Percentage reduction of daily movements on the road from Feb 10 to Feb 24** | **Percentage reduction of daily movements on train from Feb 10 to Feb 24** | **Percentage reduction of daily movements (unclassified) from Feb 10 to Mar 9** | **Percentage reduction of daily movements on plane from Feb 10 to Mar 9** | **Percentage reduction of daily movements on the road from Feb 10 to Mar 9** | **Percentage reduction of daily movements on train from Feb 10 to Mar 9** | **Percentage reduction of daily movements (unclassified) from Feb 10 to Mar 23** | **Percentage reduction of daily movements on plane from Feb 10 to Mar 23** | **Percentage reduction of daily movements on the road from Feb 10 to Mar 23** | **Percentage reduction of daily movements on train from Feb 10 to Mar 23** |
| --- | --- | --- | --- | --- | --- | --- | --- | --- | --- | --- | --- | --- | --- | --- | --- | --- | --- | --- | --- | --- | --- | --- | --- | --- | --- |
| **Italy** | 60244639 | 235244 | 390 | 19 | 35033 | 58 | 20 | 86978 | 144 | 14 | 10344 | 17 | 15 | 9 | 26 | 4 | 29 | 39 | 84 | 52 | 79 | 68 | 97 | 82 | 95 |
| **AostaValley** | 125501 | 1187 | 946 | 17 | 145 | 116 | 20 | 332 | 265 | 16 | 47 | 37 | 14 | 10 | 8 | 15 | 11 | 48 | 51 | 45 | 69 | 79 | 90 | 87 | 99 |
| Aosta | 125501 | 1187 | 946 | 17 | 145 | 116 | 20 | 332 | 265 | 16 | 47 | 37 | 14 | 10 | 8 | 15 | 11 | 48 | 51 | 45 | 69 | 79 | 90 | 87 | 99 |
| **Piedmont** | 4341375 | 30856 | 711 | 32 | 4217 | 97 | 31 | 4783 | 110 | 34 | 1861 | 43 | 45 | 13 | 16 | 6 | 45 | 38 | 79 | 52 | 82 | 68 | 100 | 82 | 96 |
| Verbano-Cusio-Ossola | 157455 | 1132 | 719 | 21 | 131 | 83 | 19 | 107 | 68 | 34 | 47 | 30 | 40 | 8 | 30 | 2 | 40 | 41 | 92 | 60 | 79 | 71 | 99 | 86 | 96 |
| Biella | 174384 | 1017 | 583 | 22 | 197 | 113 | 21 | 56 | 32 | 13 | 4 | 2 | 37 | 13 | 49 | 6 | 47 | 32 | 80 | 57 | 86 | 70 | 100 | 81 | 96 |
| Alessandria | 419037 | 4049 | 966 | 24 | 662 | 158 | 23 | 989 | 236 | 17 | 188 | 45 | 34 | 10 | 30 | 14 | 44 | 37 | 95 | 57 | 81 | 65 | 100 | 76 | 94 |
| Asti | 213216 | 1866 | 875 | 41 | 266 | 125 | 44 | 235 | 110 | 48 | 26 | 12 | 48 | 11 | 55 | 10 | 56 | 36 | 100 | 59 | 91 | 64 | 100 | 80 | 98 |
| Cuneo | 586568 | 2845 | 485 | 34 | 370 | 63 | 41 | 358 | 61 | 41 | 213 | 36 | 46 | 10 | 36 | 4 | 40 | 36 | 80 | 42 | 72 | 68 | 100 | 82 | 93 |
| Novara | 368040 | 2779 | 755 | 30 | 379 | 103 | 25 | 820 | 223 | 46 | 210 | 57 | 49 | 15 | 33 | 14 | 44 | 42 | 92 | 59 | 79 | 73 | 99 | 80 | 94 |
| Vercelli | 170296 | 1319 | 775 | 23 | 213 | 125 | 22 | 319 | 187 | 37 | 45 | 26 | 47 | 10 | 8 | 9 | 48 | 40 | 99 | 57 | 84 | 68 | 101 | 78 | 97 |
| Turin | 2252379 | 15849 | 704 | 35 | 1999 | 89 | 34 | 1899 | 84 | 36 | 1128 | 50 | 44 | 14 | 11 | 6 | 46 | 38 | 76 | 51 | 84 | 68 | 100 | 85 | 97 |
| **Liguria** | 1543127 | 9616 | 623 | 25 | 1555 | 101 | 25 | 4322 | 280 | 16 | 447 | 29 | 16 | 11 | 19 | 1 | 39 | 38 | 77 | 49 | 79 | 70 | 94 | 82 | 96 |
| La Spezia | 219196 | 885 | 404 | 17 | 156 | 71 | 20 | 454 | 207 | 15 | 38 | 17 | 15 | 17 | 22 | 1 | 22 | 43 | 86 | 50 | 73 | 76 | 99 | 85 | 96 |
| Genova | 835829 | 5611 | 671 | 28 | 936 | 112 | 26 | 2533 | 303 | 16 | 309 | 37 | 16 | 12 | 22 | 10 | 42 | 36 | 74 | 55 | 80 | 68 | 93 | 83 | 96 |
| Savona | 274183 | 1550 | 565 | 32 | 231 | 84 | 25 | 697 | 254 | 17 | 59 | 22 | 15 | 0 | 5 | 15 | 39 | 37 | 75 | 44 | 80 | 67 | 85 | 81 | 95 |
| Imperia | 213919 | 1570 | 734 | 21 | 232 | 108 | 29 | 638 | 298 | 16 | 41 | 19 | 18 | 8 | 18 | 6 | 44 | 40 | 86 | 41 | 80 | 77 | 97 | 80 | 99 |
| **Lombardy** | 10103969 | 91772 | 908 | 18 | 16621 | 164 | 18 | 44631 | 442 | 14 | 4159 | 41 | 14 | 23 | 25 | 21 | 50 | 49 | 93 | 60 | 84 | 76 | 99 | 86 | 97 |
| Monza-Brianza | 878267 | 5795 | 660 | 18 | 898 | 102 | 21 | 2660 | 303 | 15 | 225 | 26 | 15 | 23 | 33 | 19 | 42 | 49 | 94 | 60 | 80 | 77 | 100 | 87 | 97 |
| Lodi | 230607 | 3571 | 1549 | 14 | 693 | 301 | 13 | 2148 | 931 | 7 | 241 | 105 | 4 | 40 | 48 | 49 | 80 | 45 | 95 | 56 | 86 | 67 | 100 | 74 | 97 |
| Lecco | 337087 | 2839 | 842 | 16 | 470 | 139 | 17 | 1443 | 428 | 14 | 112 | 33 | 14 | 14 | 20 | 12 | 48 | 38 | 77 | 56 | 79 | 69 | 101 | 90 | 97 |
| Mantua | 411062 | 3501 | 852 | 17 | 705 | 172 | 21 | 1237 | 301 | 14 | 92 | 22 | 14 | 20 | 8 | 16 | 46 | 42 | 81 | 53 | 77 | 71 | 98 | 77 | 93 |
| Cremona | 358347 | 6504 | 1815 | 15 | 1118 | 312 | 16 | 3648 | 1018 | 11 | 342 | 95 | 11 | 32 | 49 | 33 | 60 | 49 | 97 | 57 | 78 | 73 | 100 | 79 | 91 |
| Pavia | 546515 | 5575 | 1020 | 21 | 1309 | 240 | 21 | 2629 | 481 | 15 | 235 | 43 | 15 | 25 | 28 | 23 | 46 | 42 | 95 | 52 | 74 | 70 | 99 | 77 | 92 |
| Brescia | 1268455 | 15473 | 1220 | 16 | 2679 | 211 | 17 | 8939 | 705 | 13 | 686 | 54 | 14 | 14 | 19 | 15 | 51 | 43 | 93 | 56 | 81 | 71 | 99 | 88 | 97 |
| Bergamo | 1116384 | 14394 | 1289 | 14 | 3124 | 280 | 14 | 8522 | 763 | 12 | 912 | 82 | 13 | 20 | 28 | 18 | 54 | 48 | 94 | 61 | 83 | 73 | 100 | 88 | 97 |
| Milan | 3279944 | 24499 | 747 | 24 | 4217 | 129 | 25 | 10443 | 318 | 15 | 981 | 30 | 15 | 31 | 28 | 26 | 51 | 55 | 94 | 66 | 87 | 80 | 99 | 87 | 98 |
| Sondrio | 180941 | 1596 | 882 | 32 | 212 | 117 | 25 | 563 | 311 | 16 | 52 | 29 | 15 | 1 | 33 | 2 | 19 | 42 | 82 | 54 | 63 | 70 | 95 | 90 | 93 |
| Como | 603828 | 4090 | 677 | 33 | 631 | 104 | 33 | 1273 | 211 | 16 | 146 | 24 | 17 | 18 | 11 | 15 | 43 | 45 | 93 | 58 | 81 | 77 | 99 | 88 | 97 |
| Varese | 892532 | 3935 | 441 | 37 | 565 | 63 | 43 | 1126 | 126 | 18 | 135 | 15 | 17 | 20 | 16 | 18 | 46 | 48 | 86 | 62 | 81 | 78 | 98 | 88 | 96 |
| **TrentinoSouthTyrol** | 1074819 | 7314 | 680 | 19 | 691 | 64 | 22 | 1661 | 155 | 15 | 235 | 22 | 15 | 6 | 2 | 1 | 36 | 41 | 68 | 50 | 74 | 74 | 97 | 88 | 95 |
| Trento | 542739 | 4729 | 871 | 23 | 401 | 74 | 24 | 963 | 177 | 16 | 121 | 22 | 15 | 8 | 17 | 1 | 39 | 41 | 64 | 47 | 72 | 73 | 100 | 89 | 95 |
| Bolzano | 532080 | 2585 | 486 | 17 | 290 | 55 | 20 | 698 | 131 | 15 | 114 | 21 | 15 | 4 | 14 | 1 | 35 | 40 | 72 | 51 | 75 | 74 | 95 | 87 | 96 |
| Veneto | 4907704 | 18989 | 387 | 18 | 2022 | 41 | 34 | 5239 | 107 | 15 | 798 | 16 | 14 | 19 | 12 | 15 | 51 | 37 | 89 | 54 | 86 | 69 | 99 | 83 | 97 |
| Rovigo | 233386 | 444 | 190 | 24 | 37 | 16 | 41 | 117 | 50 | 15 | 18 | 8 | 15 | 24 | 12 | 14 | 46 | 34 | 94 | 46 | 73 | 65 | 100 | 79 | 93 |
| Padua | 939672 | 3947 | 420 | 15 | 311 | 33 | 30 | 887 | 94 | 15 | 136 | 14 | 14 | 30 | 22 | 22 | 62 | 43 | 94 | 59 | 88 | 74 | 99 | 83 | 98 |
| Venezia | 851663 | 2685 | 315 | 17 | 299 | 35 | 39 | 794 | 93 | 16 | 144 | 17 | 13 | 24 | 4 | 15 | 38 | 46 | 92 | 58 | 88 | 76 | 99 | 86 | 98 |
| Treviso | 888309 | 2708 | 305 | 17 | 328 | 37 | 21 | 980 | 110 | 14 | 137 | 15 | 14 | 15 | 19 | 14 | 53 | 34 | 95 | 56 | 83 | 67 | 100 | 86 | 97 |
| Belluno | 201972 | 1166 | 577 | 23 | 112 | 55 | 44 | 210 | 104 | 17 | 16 | 8 | 15 | 3 | 51 | 11 | 22 | 33 | 87 | 48 | 70 | 70 | 100 | 91 | 93 |
| Vicenza | 862363 | 2907 | 337 | 19 | 353 | 41 | 40 | 793 | 92 | 17 | 130 | 15 | 16 | 15 | 22 | 16 | 62 | 31 | 91 | 50 | 89 | 67 | 99 | 85 | 98 |
| Verona | 930339 | 5132 | 552 | 20 | 582 | 63 | 32 | 1458 | 157 | 16 | 217 | 23 | 15 | 16 | 21 | 15 | 57 | 34 | 77 | 49 | 89 | 66 | 99 | 79 | 97 |
| **FriuliVeneziaGiulia** | 1211357 | 3301 | 273 | 17 | 357 | 29 | 26 | 881 | 73 | 15 | 98 | 8 | 15 | 17 | 15 | 9 | 47 | 38 | 82 | 44 | 77 | 71 | 99 | 82 | 95 |
| Friuli | 978081 | 1923 | 197 | 16 | 150 | 15 | 25 | 576 | 59 | 15 | 70 | 7 | 14 | 16 | 15 | 8 | 44 | 37 | 83 | 43 | 76 | 72 | 99 | 82 | 95 |
| Trieste | 233276 | 1378 | 591 | 20 | 207 | 89 | 27 | 305 | 131 | 15 | 28 | 12 | 16 | 21 | 16 | 11 | 52 | 38 | 79 | 55 | 79 | 68 | 99 | 84 | 96 |
| **EmiliaRomagna** | 4467118 | 27998 | 627 | 17 | 4373 | 98 | 19 | 10996 | 246 | 15 | 928 | 21 | 14 | 16 | 15 | 10 | 52 | 42 | 84 | 54 | 87 | 71 | 99 | 82 | 97 |
| Rimini | 339796 | 2152 | 633 | 15 | 258 | 76 | 17 | 713 | 210 | 13 | 71 | 21 | 15 | 12 | 20 | 1 | 53 | 43 | 97 | 72 | 94 | 73 | 100 | 90 | 99 |
| Forli-Cesena | 394833 | 1730 | 438 | 19 | 193 | 49 | 43 | 586 | 148 | 18 | 47 | 12 | 14 | 12 | 32 | 4 | 63 | 33 | 90 | 49 | 92 | 66 | 95 | 80 | 98 |
| Ravenna | 389634 | 1037 | 266 | 16 | 84 | 22 | 27 | 400 | 103 | 16 | 30 | 8 | 15 | 12 | 11 | 1 | 50 | 32 | 88 | 43 | 84 | 66 | 89 | 78 | 96 |
| Ferrara | 344840 | 1039 | 301 | 24 | 170 | 49 | 42 | 507 | 147 | 17 | 43 | 12 | 16 | 16 | 35 | 4 | 41 | 38 | 90 | 46 | 76 | 70 | 100 | 82 | 95 |
| Bologna | 1017806 | 5242 | 515 | 21 | 736 | 72 | 35 | 2024 | 199 | 20 | 233 | 23 | 16 | 16 | 17 | 10 | 49 | 44 | 79 | 54 | 88 | 76 | 99 | 84 | 98 |
| Modena | 707292 | 3845 | 544 | 16 | 472 | 67 | 19 | 1107 | 157 | 15 | 84 | 12 | 14 | 16 | 46 | 13 | 53 | 42 | 91 | 57 | 89 | 73 | 99 | 83 | 98 |
| Reggio Emilia | 531751 | 4896 | 921 | 18 | 583 | 110 | 23 | 1535 | 289 | 15 | 60 | 11 | 11 | 19 | 13 | 13 | 50 | 45 | 93 | 56 | 86 | 73 | 99 | 82 | 96 |
| Parma | 453930 | 3634 | 801 | 15 | 912 | 201 | 16 | 2171 | 478 | 13 | 98 | 22 | 11 | 20 | 19 | 15 | 52 | 48 | 86 | 58 | 85 | 70 | 100 | 78 | 96 |
| Piacenza | 287236 | 4423 | 1540 | 16 | 965 | 336 | 16 | 1953 | 680 | 12 | 262 | 91 | 13 | 25 | 32 | 20 | 69 | 44 | 94 | 50 | 85 | 68 | 100 | 76 | 94 |
| **Tuscany** | 3722729 | 9570 | 257 | 18 | 1076 | 29 | 31 | 2929 | 79 | 16 | 201 | 5 | 24 | 3 | 10 | 5 | 9 | 37 | 65 | 48 | 77 | 66 | 98 | 82 | 96 |
| Prato | 258152 | 521 | 202 | 19 | 49 | 19 | 24 | 171 | 66 | 16 | 3 | 1 | 114 | 4 | 21 | 7 | 33 | 40 | 69 | 48 | 82 | 75 | 100 | 86 | 98 |
| Grosseto | 220785 | 378 | 171 | 16 | 24 | 11 | 22 | 84 | 38 | 15 | 7 | 3 | 24 | 2 | 77 | 5 | 1 | 26 | 90 | 43 | 69 | 57 | 83 | 77 | 90 |
| Siena | 266238 | 422 | 159 | 15 | 32 | 12 | 22 | 95 | 36 | 16 | 6 | 2 | 16 | 2 | 13 | 10 | 12 | 29 | 53 | 50 | 81 | 53 | 100 | 81 | 96 |
| Arezzo | 341766 | 675 | 198 | 20 | 47 | 14 | 41 | 112 | 33 | 22 | 8 | 2 | 42 | 2 | 30 | 6 | 12 | 28 | 45 | 50 | 75 | 54 | 100 | 83 | 95 |
| Pisa | 422310 | 901 | 213 | 17 | 90 | 21 | 21 | 280 | 66 | 16 | 12 | 3 | 33 | 4 | 9 | 2 | 12 | 41 | 56 | 48 | 79 | 70 | 99 | 82 | 97 |
| Livorno | 333509 | 456 | 137 | 17 | 59 | 18 | 27 | 145 | 43 | 15 | 6 | 2 | 23 | 6 | 8 | 6 | 5 | 40 | 61 | 40 | 71 | 72 | 75 | 78 | 95 |
| Firenze | 1004298 | 3118 | 310 | 24 | 390 | 39 | 40 | 1062 | 106 | 18 | 52 | 5 | 40 | 2 | 10 | 1 | 11 | 38 | 75 | 54 | 80 | 65 | 100 | 86 | 98 |
| Pistoia | 293059 | 728 | 248 | 18 | 76 | 26 | 17 | 302 | 103 | 15 | 15 | 5 | 16 | 8 | 17 | 3 | 10 | 39 | 76 | 44 | 74 | 66 | 99 | 84 | 96 |
| Lucca | 388678 | 1320 | 340 | 16 | 141 | 36 | 30 | 365 | 94 | 15 | 43 | 11 | 23 | 1 | 56 | 19 | 19 | 42 | 69 | 43 | 74 | 72 | 97 | 83 | 97 |
| Massa-Carrara | 193934 | 1051 | 542 | 17 | 168 | 87 | 26 | 313 | 161 | 17 | 49 | 25 | 20 | 8 | 44 | 0 | 20 | 44 | 82 | 56 | 87 | 74 | 92 | 85 | 98 |
| **Umbria** | 880285 | 1420 | 161 | 15 | 76 | 9 | 20 | 360 | 41 | 15 | 77 | 9 | 15 | 1 | 6 | 8 | 12 | 30 | 82 | 50 | 69 | 60 | 99 | 81 | 92 |
| Terni | 224882 | 409 | 182 | 16 | 29 | 13 | 18 | 110 | 49 | 15 | 23 | 10 | 16 | 0 | 56 | 8 | 10 | 32 | 93 | 46 | 73 | 63 | 100 | 80 | 96 |
| Perugia | 655403 | 1011 | 154 | 14 | 47 | 7 | 22 | 250 | 38 | 14 | 54 | 8 | 14 | 1 | 13 | 9 | 12 | 29 | 78 | 52 | 67 | 59 | 99 | 82 | 89 |
| **Marche** | 1518400 | 6573 | 433 | 16 | 964 | 63 | 18 | 2121 | 140 | 14 | 265 | 17 | 13 | 2 | 36 | 2 | 30 | 36 | 79 | 56 | 78 | 68 | 91 | 85 | 96 |
| Fermo | 173004 | 484 | 280 | 17 | 64 | 37 | 17 | 74 | 43 | 14 | 9 | 5 | 14 | 2 | 22 | 0 | 25 | 40 | 100 | 54 | 87 | 73 | 100 | 87 | 98 |
| Ascoli Piceno | 206363 | 238 | 115 | 15 | 11 | 5 | 20 | 38 | 18 | 14 | 4 | 2 | 13 | 1 | 92 | 4 | 15 | 39 | 100 | 50 | 81 | 71 | 100 | 83 | 98 |
| Macerata | 312146 | 1167 | 374 | 18 | 145 | 46 | 23 | 257 | 82 | 15 | 27 | 9 | 15 | 3 | 5 | 0 | 25 | 36 | 100 | 52 | 76 | 69 | 95 | 87 | 97 |
| Ancona | 469750 | 1803 | 384 | 16 | 216 | 46 | 21 | 611 | 130 | 14 | 48 | 10 | 13 | 3 | 39 | 5 | 28 | 33 | 68 | 57 | 73 | 64 | 87 | 84 | 94 |
| Pesaro | 357137 | 2881 | 807 | 15 | 528 | 148 | 17 | 1141 | 319 | 13 | 177 | 50 | 12 | 2 | 27 | 4 | 40 | 38 | 100 | 64 | 87 | 68 | 99 | 86 | 98 |
| **Lazio** | 5865544 | 7771 | 132 | 17 | 867 | 15 | 35 | 2755 | 47 | 16 | 331 | 6 | 14 | 1 | 22 | 2 | 12 | 41 | 72 | 45 | 77 | 67 | 84 | 81 | 96 |
| Frosinone | 485241 | 659 | 136 | 16 | 82 | 17 | 19 | 234 | 48 | 14 | 21 | 4 | 12 | 3 | 63 | 4 | 8 | 29 | 96 | 47 | 81 | 50 | 99 | 80 | 98 |
| Latina | 576655 | 603 | 105 | 16 | 43 | 7 | 24 | 222 | 38 | 15 | 28 | 5 | 12 | 0 | 3 | 7 | 5 | 27 | 90 | 44 | 77 | 50 | 100 | 79 | 97 |
| Roma | 4333274 | 5654 | 130 | 18 | 669 | 15 | 38 | 2161 | 50 | 17 | 262 | 6 | 15 | 2 | 22 | 0 | 13 | 45 | 71 | 46 | 77 | 72 | 84 | 82 | 96 |
| Rieti | 154232 | 408 | 265 | 16 | 44 | 29 | 27 | 59 | 38 | 17 | 8 | 5 | 15 | 0 | 83 | 8 | 8 | 28 | 70 | 43 | 77 | 54 | 97 | 79 | 96 |
| Viterbo | 316142 | 447 | 141 | 17 | 29 | 9 | 44 | 79 | 25 | 17 | 12 | 4 | 14 | 1 | 25 | 8 | 6 | 28 | 71 | 44 | 70 | 53 | 89 | 79 | 96 |
| **Abruzzo** | 1305770 | 3256 | 249 | 17 | 458 | 35 | 28 | 1177 | 90 | 16 | 129 | 10 | 15 | 1 | 48 | 9 | 15 | 36 | 91 | 52 | 82 | 66 | 99 | 86 | 97 |
| Chieti | 383189 | 818 | 213 | 19 | 121 | 32 | 35 | 306 | 80 | 19 | 36 | 9 | 15 | 1 | 48 | 5 | 10 | 33 | 89 | 53 | 86 | 61 | 98 | 85 | 98 |
| Pescara | 318678 | 1578 | 495 | 18 | 254 | 80 | 35 | 569 | 179 | 16 | 66 | 21 | 14 | 2 | 54 | 4 | 20 | 39 | 89 | 56 | 82 | 67 | 99 | 86 | 98 |
| Teramo | 307412 | 635 | 207 | 16 | 74 | 24 | 18 | 210 | 68 | 15 | 14 | 5 | 14 | 2 | 47 | 6 | 15 | 41 | 100 | 50 | 80 | 71 | 100 | 83 | 96 |
| L'Aquila | 296491 | 225 | 76 | 16 | 9 | 3 | 18 | 92 | 31 | 13 | 13 | 4 | 17 | 2 | 23 | 16 | 7 | 34 | 101 | 50 | 76 | 65 | 100 | 88 | 97 |
| **Molise** | 302265 | 427 | 141 | 22 | 21 | 7 | 21 | 99 | 33 | 15 | 10 | 3 | 12 | 1 | 50 | 10 | 13 | 29 | 94 | 52 | 77 | 59 | 100 | 83 | 94 |
| Isernia | 83586 | 62 | 74 | 21 | 5 | 6 | 40 | 15 | 18 | 21 | 1 | 1 | 13 | 3 | 61 | 16 | 4 | 30 | 100 | 51 | 72 | 63 | 100 | 84 | 94 |
| Campobasso | 218679 | 365 | 167 | 23 | 16 | 7 | 19 | 84 | 38 | 15 | 9 | 4 | 11 | 0 | 75 | 7 | 16 | 29 | 92 | 53 | 79 | 58 | 100 | 83 | 94 |
| **Campania** | 5785861 | 4721 | 82 | 18 | 475 | 8 | 22 | 1055 | 18 | 15 | 123 | 2 | 15 | 6 | 27 | 5 | 12 | 39 | 86 | 49 | 78 | 67 | 99 | 80 | 95 |
| Salerno | 1092779 | 696 | 64 | 17 | 64 | 6 | 20 | 168 | 15 | 15 | 15 | 1 | 15 | 4 | 42 | 5 | 16 | 34 | 90 | 50 | 78 | 63 | 97 | 80 | 96 |
| Avellino | 413926 | 534 | 129 | 17 | 59 | 14 | 18 | 158 | 38 | 14 | 14 | 3 | 13 | 4 | 47 | 3 | 24 | 31 | 95 | 52 | 84 | 62 | 100 | 82 | 99 |
| Napoli | 3082905 | 2620 | 85 | 18 | 293 | 10 | 24 | 557 | 18 | 16 | 89 | 3 | 16 | 6 | 17 | 5 | 8 | 43 | 83 | 50 | 76 | 70 | 99 | 82 | 94 |
| Benevento | 274080 | 208 | 76 | 19 | 16 | 6 | 33 | 54 | 20 | 15 | 2 | 1 | 12 | 4 | 56 | 5 | 14 | 28 | 91 | 51 | 78 | 57 | 100 | 81 | 97 |
| Caserta | 922171 | 663 | 72 | 18 | 43 | 5 | 18 | 118 | 13 | 16 | 3 | 0 | 14 | 8 | 57 | 5 | 16 | 42 | 92 | 44 | 80 | 71 | 99 | 78 | 96 |
| **Apulia** | 4008296 | 4496 | 112 | 20 | 546 | 14 | 32 | 1699 | 42 | 17 | 270 | 7 | 18 | 3 | 45 | 5 | 5 | 36 | 89 | 52 | 64 | 66 | 99 | 80 | 88 |
| BAT | 388390 | 380 | 98 | 23 | 63 | 16 | 44 | 158 | 41 | 33 | 31 | 8 | 37 | 1 | 49 | 3 | 5 | 32 | 91 | 51 | 66 | 64 | 100 | 77 | 89 |
| Lecce | 791122 | 521 | 66 | 16 | 76 | 10 | 19 | 234 | 30 | 15 | 26 | 3 | 19 | 0 | 38 | 8 | 9 | 40 | 89 | 57 | 63 | 72 | 100 | 85 | 87 |
| Brindisi | 390456 | 658 | 169 | 23 | 66 | 17 | 36 | 180 | 46 | 19 | 15 | 4 | 16 | 3 | 41 | 2 | 1 | 38 | 86 | 53 | 59 | 68 | 99 | 79 | 82 |
| Taranto | 572772 | 281 | 49 | 18 | 33 | 6 | 43 | 131 | 23 | 16 | 19 | 3 | 21 | 7 | 47 | 1 | 1 | 31 | 94 | 52 | 55 | 61 | 100 | 78 | 82 |
| Bari | 1249246 | 1488 | 119 | 20 | 152 | 12 | 27 | 535 | 43 | 16 | 96 | 8 | 16 | 4 | 48 | 5 | 6 | 41 | 89 | 53 | 66 | 70 | 99 | 82 | 89 |
| Foggia | 616310 | 1168 | 190 | 24 | 156 | 25 | 31 | 461 | 75 | 18 | 83 | 13 | 17 | 2 | 53 | 10 | 8 | 27 | 89 | 46 | 67 | 58 | 94 | 74 | 89 |
| Basilicata | 556934 | 366 | 66 | 17 | 30 | 5 | 29 | 120 | 22 | 18 | 30 | 5 | 15 | 1 | 25 | 3 | 11 | 30 | 81 | 51 | 59 | 62 | 99 | 79 | 82 |
| Matera | 195998 | 193 | 98 | 19 | 9 | 5 | 45 | 58 | 30 | 24 | 13 | 7 | 16 | 4 | 5 | 8 | 22 | 26 | 76 | 49 | 52 | 54 | 99 | 75 | 74 |
| Potenza | 360936 | 173 | 48 | 15 | 21 | 6 | 23 | 62 | 17 | 16 | 17 | 5 | 14 | 1 | 59 | 1 | 1 | 33 | 91 | 52 | 65 | 66 | 100 | 83 | 90 |
| **Calabria** | 1924701 | 1193 | 62 | 17 | 97 | 5 | 23 | 356 | 18 | 16 | 50 | 3 | 15 | 2 | 37 | 1 | 2 | 37 | 85 | 51 | 56 | 64 | 99 | 76 | 82 |
| Vibo Valentia | 157469 | 85 | 54 | 15 | 5 | 3 | 18 | 22 | 14 | 16 | 5 | 3 | 14 | 2 | 32 | 5 | 2 | 32 | 88 | 46 | 29 | 59 | 100 | 70 | 53 |
| Crotone | 170718 | 118 | 69 | 14 | 7 | 4 | 16 | 34 | 20 | 14 | 8 | 5 | 14 | 1 | 13 | 1 | 8 | 37 | 78 | 52 | 58 | 68 | 99 | 78 | 85 |
| Reggio Calabria | 541278 | 315 | 58 | 16 | 19 | 4 | 16 | 96 | 18 | 15 | 13 | 2 | 15 | 1 | 41 | 0 | 4 | 38 | 85 | 49 | 57 | 64 | 98 | 75 | 86 |
| Catanzaro | 354851 | 204 | 57 | 17 | 32 | 9 | 30 | 100 | 28 | 18 | 11 | 3 | 18 | 3 | 40 | 2 | 5 | 38 | 86 | 51 | 60 | 65 | 99 | 77 | 84 |
| Cosenza | 700385 | 471 | 67 | 23 | 34 | 5 | 32 | 104 | 15 | 16 | 13 | 2 | 15 | 4 | 37 | 0 | 4 | 37 | 89 | 52 | 53 | 64 | 100 | 78 | 78 |
| **Sicily** | 4968410 | 3054 | 61 | 17 | 309 | 6 | 28 | 1132 | 23 | 16 | 201 | 4 | 16 | 3 | 30 | 4 | 9 | 36 | 83 | 49 | 61 | 65 | 99 | 79 | 88 |
| Siracusa | 397037 | 334 | 84 | 22 | 45 | 11 | 44 | 165 | 42 | 20 | 26 | 7 | 25 | 2 | 28 | 14 | 4 | 34 | 84 | 46 | 60 | 67 | 100 | 78 | 81 |
| Ragusa | 321215 | 91 | 28 | 20 | 6 | 2 | 20 | 26 | 8 | 16 | 5 | 2 | 16 | 1 | 45 | 9 | 4 | 32 | 91 | 43 | 63 | 60 | 100 | 75 | 80 |
| Catania | 1104974 | 777 | 70 | 17 | 97 | 9 | 28 | 336 | 30 | 17 | 84 | 8 | 16 | 3 | 38 | 3 | 1 | 37 | 86 | 49 | 59 | 63 | 100 | 78 | 75 |
| Enna | 162368 | 431 | 265 | 16 | 35 | 22 | 22 | 126 | 78 | 15 | 15 | 9 | 15 | 2 | 7 | 9 | 27 | 32 | 82 | 40 | 89 | 63 | 100 | 67 | 99 |
| Caltanissetta | 260779 | 187 | 72 | 19 | 13 | 5 | 21 | 55 | 21 | 16 | 11 | 4 | 14 | 3 | 39 | 5 | 7 | 34 | 84 | 51 | 61 | 66 | 101 | 83 | 93 |
| Agrigento | 429611 | 137 | 32 | 16 | 13 | 3 | 19 | 38 | 9 | 15 | 4 | 1 | 15 | 0 | 20 | 4 | 11 | 39 | 70 | 55 | 65 | 71 | 96 | 82 | 88 |
| Messina | 620721 | 459 | 74 | 16 | 57 | 9 | 29 | 206 | 33 | 16 | 19 | 3 | 16 | 1 | 36 | 0 | 9 | 41 | 85 | 47 | 54 | 65 | 100 | 75 | 87 |
| Palermo | 1243328 | 499 | 40 | 18 | 37 | 3 | 30 | 140 | 11 | 16 | 24 | 2 | 14 | 7 | 33 | 2 | 14 | 36 | 82 | 54 | 61 | 63 | 100 | 83 | 89 |
| Trapani | 428377 | 139 | 32 | 16 | 6 | 1 | 23 | 40 | 9 | 15 | 13 | 3 | 14 | 3 | 17 | 0 | 14 | 36 | 81 | 53 | 59 | 68 | 99 | 82 | 90 |
| **Sardinia** | 1630474 | 1364 | 84 | 17 | 133 | 8 | 25 | 330 | 20 | 16 | 84 | 5 | 18 | 3 | 26 | 7 | 4 | 35 | 83 | 47 | 49 | 62 | 99 | 79 | 72 |
| Sud Sardegna | 347005 | 119 | 34 | 16 | 16 | 5 | 33 | 40 | 12 | 17 | 9 | 3 | 46 | 2 | 11 | 7 | 3 | 32 | 72 | 44 | 44 | 60 | 99 | 76 | 60 |
| Oristano | 156078 | 69 | 44 | 24 | 5 | 3 | 42 | 21 | 13 | 16 | 5 | 3 | 17 | 2 | 117 | 25 | 37 | 36 | 80 | 52 | 50 | 61 | 100 | 81 | 80 |
| Cagliari | 430914 | 231 | 54 | 20 | 16 | 4 | 35 | 51 | 12 | 16 | 9 | 2 | 14 | 4 | 36 | 2 | 1 | 41 | 85 | 48 | 50 | 67 | 99 | 78 | 73 |
| Nuoro | 206843 | 94 | 45 | 15 | 10 | 5 | 20 | 27 | 13 | 16 | 6 | 3 | 15 | 5 | 11 | 8 | 2 | 28 | 89 | 52 | 62 | 57 | 100 | 81 | 81 |
| Sassari | 489634 | 851 | 174 | 17 | 86 | 18 | 23 | 191 | 39 | 16 | 55 | 11 | 19 | 4 | 38 | 3 | 2 | 33 | 83 | 46 | 53 | 60 | 99 | 80 | 84 |

**Supplemental Figure S1:** Cumulative incidence of health outcomes in the early phase of the first COVID-19 wave in Italy (February 24 through March 9, 2020) and percentage of mobile phone movement reductions compared with Monday, Feb 10, 2020.

**
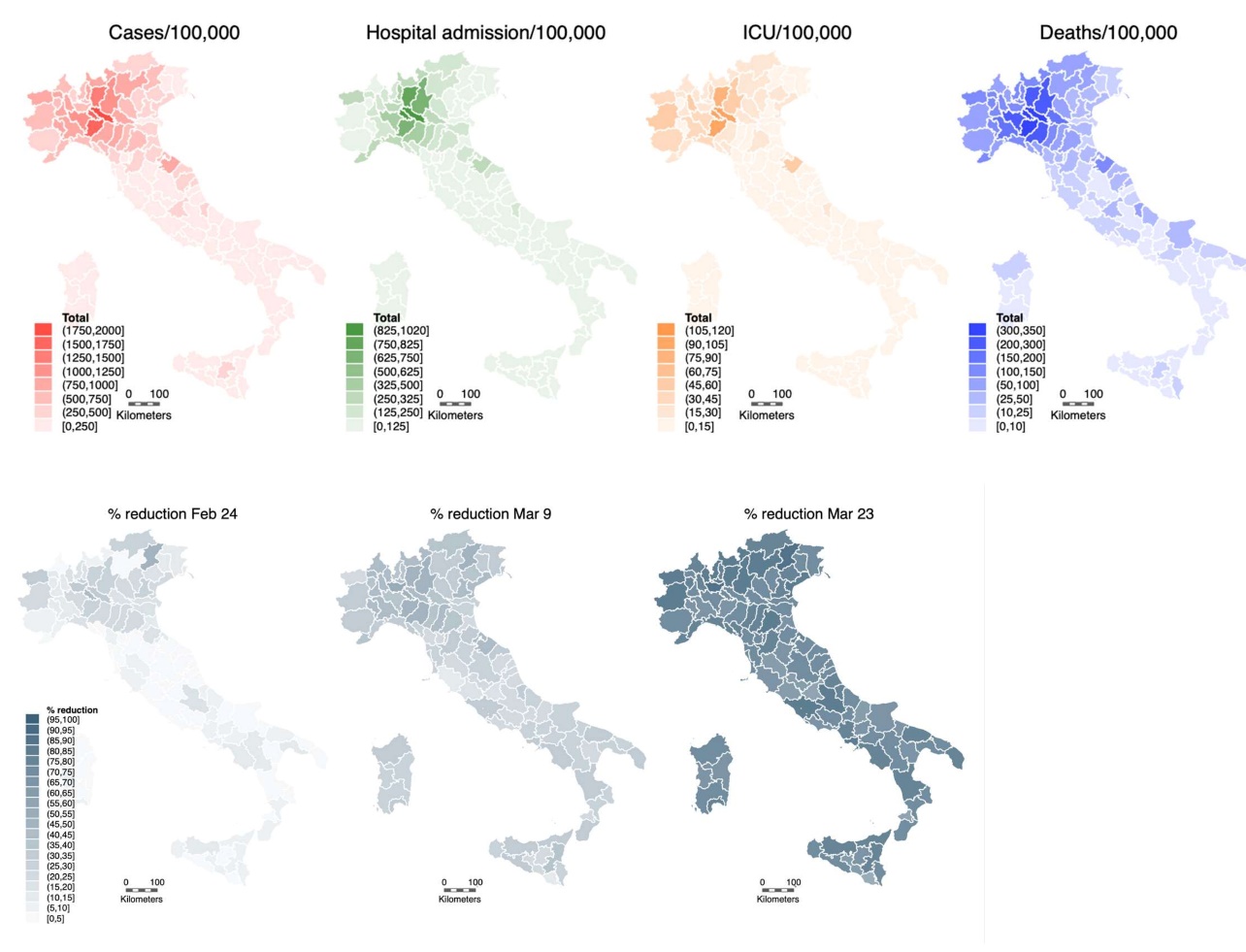
**

**Supplemental Figure S2.** Days until the peak of the COVID-19 endpoints and percent reduction of people movements for provinces in Italy in provinces with cumulative deaths/10^5^ ≥200. The solid line shows the results of the spline analysis, while the size of the bubbles is proportional to the cumulative number of cases at March 8, 2020.

**Supplemental Figure S3.** Days until the peak of the COVID-19 endpoints and percent reduction of people movements for provinces in Italy in a) the entire dataset, b) for age <70, c) in provinces with cumulative deaths/10^5^ ≥50. The solid lines correspond to the models adjusted for meteorological factors, PM_10_, old age index, population and single-family homes, while the dashed ones to the models not adjusted for air pollution, for meteorological factors and for air pollution and meteorological factors.


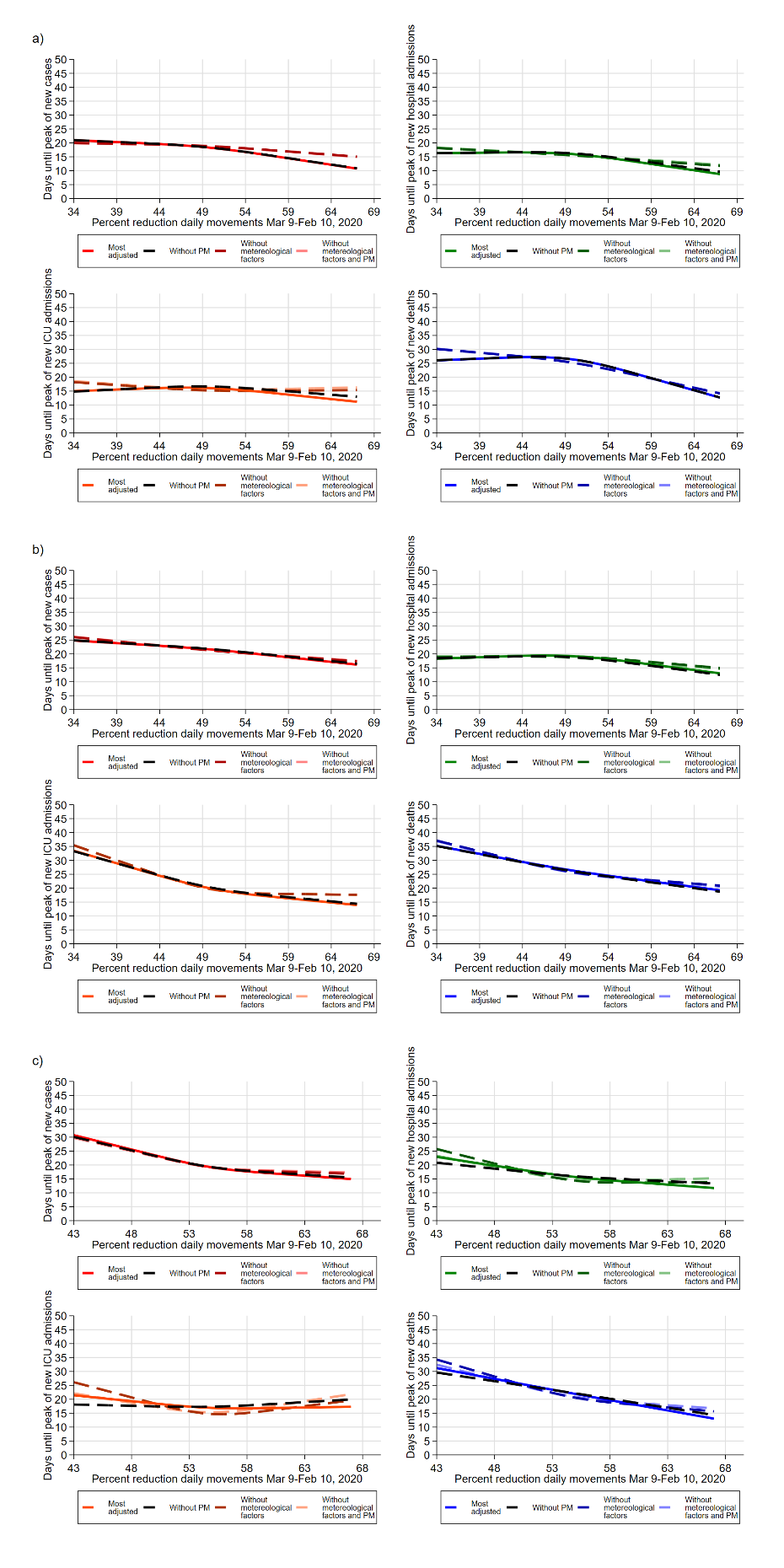


**Supplemental Figure S4.** Days until the peak of the COVID-19 endpoints and percent reduction of people movements for provinces in Italy: a) road movements; b) train movements; c) plane movements. The solid lines correspond to the models adjusted for meteorological factors, PM_10_, population, old age index, single-family homes and for movement reductions related to the other two means of transport, while the dashed lines correspond to the original analysis, not adjusted for the other two means of transport.

**
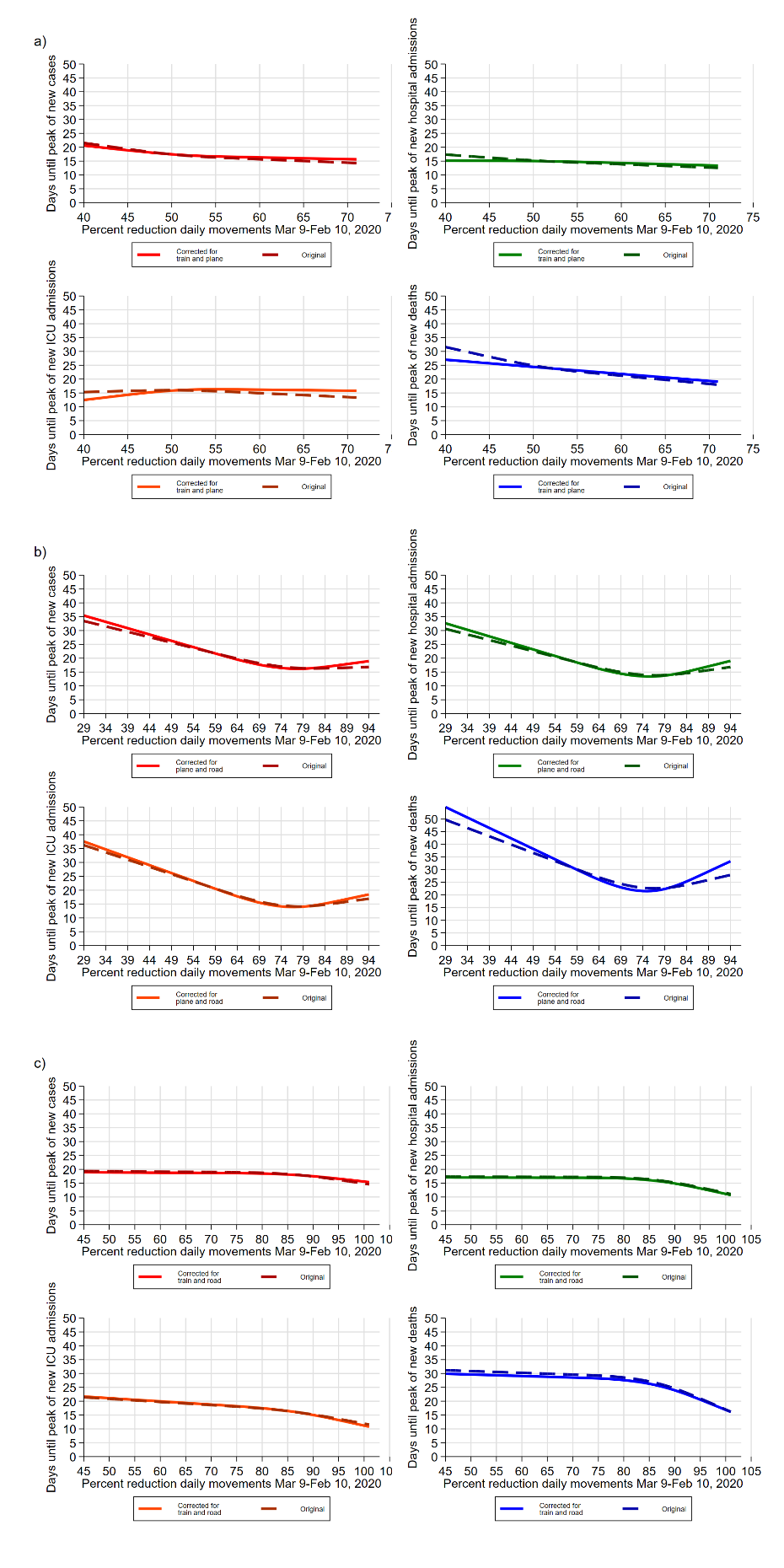
**
